# Integrated multi-omics profiling of immune microenvironment and drug resistance signatures for precision prognosis in prostate cancer

**DOI:** 10.20517/cdr.2025.47

**Published:** 2025-06-25

**Authors:** Chao Li, Longxiang Wu, Bowen Zhong, Yu Gan, Lei Zhou, Shuo Tan, Weibin Hou, Kun Yao, Bingzhi Wang, Zhenyu Ou, Shengwang Zhang, Wei Xiong

**Affiliations:** ^1^Department of Urology, The Third Xiangya Hospital, Central South University, Changsha 410013, Hunan, China.; ^2^Department of Urology, Xiangya Hospital, Central South University, Changsha 410008, Hunan, China.; ^3^Department of Radiology, The Third Xiangya Hospital, Central South University, Changsha 410013, Hunan, China.; ^#^Authors contributed equally.

**Keywords:** Tumor microenvironment, prostate cancer, drug resistance, immunotherapy, prognostic model

## Abstract

**Introduction:** Prostate cancer (PCa) continues to be a significant cause of mortality among men, with treatment resistance often influenced by the complexity of the tumor microenvironment (TME). This study aims to develop an immune-centric prognostic model that correlates TME dynamics, genomic instability, and the heterogeneity of drug resistance in PCa.

**Methods:** Multi-omics data from The Cancer Genome Atlas (TCGA) and Gene Expression Omnibus (GEO) databases were integrated, encompassing transcriptomic profiles of 554 TCGA-PRAD samples and 329 external validation samples. Immune cell infiltration was assessed using CIBERSORT and ESTIMATE. Weighted gene co-expression network analysis (WGCNA) was employed to identify immune-related modules. Single-cell RNA sequencing (ScRNA-seq) of 835 PCa cells uncovered subtype-specific resistance patterns. Prognostic models were constructed using least absolute shrinkage and selection operator (LASSO) regression and subsequently validated experimentally in PCa cell lines.

**Results:** Two immune subtypes were identified: high-risk subgroups displayed TP53 mutations, increased tumor mutation burden (TMB), and enriched energy metabolism pathways. ScRNA-seq delineated three PCa cell clusters, with high-risk subtypes being sensitive to bendamustine/dacomitinib and resistant to apalutamide/neratinib. A 10-gene prognostic model (e.g., MUC5B, TREM1) categorized patients into high/low-risk groups with distinct survival outcomes (log-rank *P* < 0.0001). Validation in external datasets confirmed the robust predictive accuracy (AUC: 0.854-0.889). Experimental assays verified subtype-specific drug responses and dysregulation of key model genes.

**Discussion:** This study establishes a TME-driven prognostic framework that connects immune heterogeneity, genomic instability, and therapeutic resistance in PCa. By pinpointing metabolic dependencies and subtype-specific vulnerabilities, our findings provide actionable strategies to circumvent treatment failure, such as targeting energy metabolism or tailoring therapies based on resistance signatures.

## INTRODUCTION

Prostate cancer (PCa) is a common solid malignancy among men worldwide^[1]^. Despite considerable advancements in the diagnosis and treatment, the incidence and mortality rates of metastatic PCa remain high, and accurate prognostication remains a challenge.

The tumor microenvironment (TME) includes tumor stromal cells such as immune cells, fibroblasts, and endothelial cells, in addition to numerous signaling molecules such as cytokines and chemokines^[2]^. In PCa, the TME is exceptionally complex, and the interactions between these different cellular components profoundly influence disease progression and treatment outcomes^[3]^. In recent years, the introduction of high-throughput -omics technologies and bioinformatics methods has advanced our understanding of the interplay between PCa and the immune system^[4]^. These tools enable a comprehensive characterization of the immune landscape within tumors and the identification of immune-related biomarkers with prognostic significance^[5,6]^.

Elucidating the immune environment in PCa is not only crucial for accurate prognosis prediction but also for the development of therapeutic interventions targeting the immune system. Immune system-based therapies have shown great promise in treating PCa, given their successful application in other malignancies^[7]^. Nonetheless, owing to the heterogeneity of PCa and the intricate nature of the immune microenvironment, the clinical application of immunotherapy in PCa remains challenging, creating a need to establish robust immune environment-related prognostic models that can identify patients most likely to benefit from immunotherapeutic approaches. Such a model may enhance prognostic accuracy and provide new avenues for personalized treatment interventions.

In this study, we aimed to use bioinformatics techniques to examine the immune landscape of PCa, leveraging The Cancer Genome Atlas (TCGA) dataset to identify TME subtypes. We aimed to identify the pivotal immune-related genes and pathways linked to prognosis within the high-risk subtype and to construct a novel immune-related prognostic prediction model. By integrating multiomics data and clinical information, we aimed to document the TME heterogeneity and its clinically relevant characteristics. In addition, we aimed to use this model to identify immune-related treatment targets. This study may advance our understanding of the TME in PCa and may support clinical practice and treatment selection.

## METHODS

### Data collection and preprocessing

Count expression profiles and corresponding clinical data for prostate adenocarcinoma (PRAD) were downloaded from the TCGA (https://www.cancer.gov)^[8]^. This dataset contained 554 samples, including 52 PRAD-adjacent “normal” and 502 PRAD cancer samples. In addition, PCa tissue transcriptome sequencing data were collected from the Gene Expression Omnibus (GEO) database (https://www.ncbi.nlm.nih.gov/geo)^[9]^, including three datasets with accession numbers GSE46602^[10]^, GSE70769^[11]^, and GSE116918^[12]^. All these datasets were derived from *Homo sapiens* and were related to PCa. GSE46602 contained 50 samples, including 36 cancer samples and 14 adjacent “normal” samples. Only cancer samples were included in the analysis and the sequencing platform used was GPL570. GSE70769 included 94 cancer samples with no adjacent “normal” samples, of which 45 samples had matching survival data. The sequencing platform used was GPL10558. GSE116918 contained 248 cancer samples with no adjacent “normal” samples, and all had survival data with the sequencing platform GPL25318. These datasets were neither merged nor subjected to batch effect correction. Instead, the prognostic model was applied to each dataset separately for external validation. This approach ensures that model performance can be robustly evaluated across different platforms, avoiding additional technical variations introduced by data integration. In total, 329 cancer samples with survival data were used for model validation.

In addition, single-cell RNA sequencing (scRNA-seq) data for PCa were obtained from the literature^[13]^, and included 2,170 cells sequenced on the Illumina NexteraXT platform. Based on the previously reported cell labels^[13]^, 835 PCa cells were extracted for single-cell level validation.

### Tumor immune infiltration analysis

The immune scores and infiltration levels of 22 immune cell types in PCa samples from TCGA were calculated using the R package estimate^[14]^ and CIBERSORT^[15]^. The functions filterCommonGenes and estimateScore in the estimate package were used with the default parameters. In the CIBERSORT analysis, we determined the infiltration scores of 22 immune cell types for each sample. These scores were calculated using the LM22 background gene set provided by CIBERSORT^[15]^, with the perm parameter set to 500 and the other parameters set to default values.

Subsequently, unsupervised clustering was performed on all PCa samples based on immune scores and immune cell infiltration matrix. To determine the optimal number of clusters, the fviz_nbclust function in the R package factoextra was used, with the average silhouette width as the evaluation metric^[16]^. The optimal cluster number (k = 2) was determined by maximizing the average silhouette width across candidate cluster numbers. Samples were subsequently partitioned using k-means clustering and visualized via principal component analysis (PCA).

The infiltration levels of the 22 immune cell types in the different sample groups were visualized in box plots using ggplot2^[17]^ and ggpubr^[18]^ R packages. Statistical significance was assessed using the Wilcoxon rank-sum test. Heatmaps and stacked bar plots were used to visualize the immune scores and the distribution of immune cell infiltration levels, respectively.

### Comparisons of immune features

To compare the immune characteristics between the groups, we used correlation analysis of the 22 immune cell types in the TCGA samples using the corrplot R package^[19]^. PD-1 and PD-L1 are two prominent immune checkpoint genes targeted in immunotherapy, and we used the violin and box plots to examine their expression levels in different sample groups. Subsequently, the analysis of differential gene expression between the groups was conducted using the DESeq2 package^[20]^, using adjusted *P*-values of < 0.05 as a significance threshold, and an absolute log2-fold change (log2FC) greater than 1. Significantly differentially expressed genes were visualized using volcano plots and heatmaps with the ggplot2^[17]^ and pheatmap^[21]^ R packages.

Based on previous studies, *CTLA4*, *HAVCR2*, *IDO1*, and *LAG3* are categorized as immune checkpoint-related markers, whereas *CD8A*, *CXCL10*, *CXCL9*, *GZMA*, *GZMB*, *IFNG*, *PRF1*, *TBX2*, and *TNF* are designated as immune-related markers^[22]^. The expression levels of these two categories of genes were examined in different sample groups, and their statistical significance was ascertained using the Wilcoxon rank-sum test.

### Weighted gene co-expression network analysis

Weighted gene co-expression network analysis (WGCNA) was used to identify the gene modules associated with the sample grouping labels of differentially expressed genes. This entails grouping genes with analogous expression patterns and investigating their associations with particular traits or phenotypes^[23]^. The pickSoftThreshold function from the R package WGCNA was used to select the optimal soft threshold^[24]^, and to calculate both the scale-free topology fit index and mean connectivity across a range of candidate soft-thresholding powers. The optimal power of 3 was determined by considering the point where the scale-free topology fit index first exceeded 0.8, and the inflection point in the corresponding curve, thereby balancing the network’s scale-free property with mean connectivity preservation. Gene module visualization was performed using the plotDendroAndColors function and correlations between gene modules were visualized using a correlation heatmap. To identify the gene modules associated with TCGA sample grouping labels, the results were visualized using the Labeled Heatmap function.

Moreover, for the purpose of examining the biological functions linked to the correlated gene modules, gene enrichment analysis was conducted using Gene Ontology (GO) and Kyoto Encyclopedia of Genes and Genomes (KEGG) methods. The GO enrichment analysis is commonly used to study the large-scale functional enrichment of genes in different dimensions and at different levels^[25]^. The KEGG database is frequently used to store information related to genomes, pathways, diseases, and drugs^[26]^. The R package clusterProfiler^[27,28]^ was used to annotate gene functions and perform the KEGG pathway enrichment on genes in the associated gene modules, with a significance threshold set at corrected *P*-values of < 0.05.

### Construction and validation of prognostic prediction models

A prognostic model was constructed using univariate Cox and least absolute shrinkage and selection operator (LASSO) regression analyses based on the gene modules mentioned above, and the validity and predictive efficacy of the model were verified using three external datasets.

The survival function in the R package was used to perform univariate Cox regression^[29]^, with a significance threshold set at *P-*values of < 0.05. Subsequently, the genes that met the statistical threshold were used as inputs for the LASSO regression models using the glmnet^[30]^ function in the R package. The cv.glmnet function was used for model construction, with the family parameter set to Cox and alpha set to 1. Genes with coefficient values of zero were removed to establish the final model. Using the constructed models, risk scores were allocated to the TCGA PRAD dataset. The surv_cutpoint function from the survminer^[31]^ R package was used to select the optimal grouping threshold by dividing all samples into high- and low-risk groups. Subsequently, stratified survival analysis was conducted on the datasets from these groups, and the Kaplan-Meier (KM) survival curves were generated to evaluate the model’s predictive performance. The receiver operating characteristic (ROC) curves were plotted to predict 1-, 3-, and 5-year survival rates in the original dataset using the R package timeROC^[32]^. In addition, the same risk-scoring process was applied to three external datasets, GSE46602^[10]^, GSE70769^[11]^, and GSE116918^[12]^, to assess the model predictive performance by plotting the KM survival curves.

### Biological function analysis in high- and low-risk groups

To further investigate the variations in biological functions between the high- and low-risk groups within the GEO dataset, we conducted differential gene expression analysis using the R package DESeq2^[20]^. We applied a significance threshold of corrected *P*-values of < 0.05 and an absolute log2FC exceeding 1. The results of the differential gene expression analysis are represented using volcano plots and heatmaps.

Subsequently, the R package clusterProfiler^[27,28]^ was used to perform GO and KEGG enrichment analyses on significantly differentially expressed genes, employing corrected *P*-values of < 0.05. Enrichment analysis results are presented as bar and bubble plots.

### Gene mutation feature analysis

To reveal disparities in genomic characteristics between the high- and low-risk group samples, we performed gene-level mutation analysis and assessed the tumor mutation burden (TMB).

First, the function tcga_load in the R package for TCGA mutations (https://github.com/PoisonAlien/TCGAmutations) was used to obtain gene mutation data for PRAD, and the R package maftools^[33]^ was employed for mutation data analysis. The Oncoplot function was used to create waterfall plots depicting the gene mutations. The TMB was calculated using the tmb function. To assess any differences in TMB between the high- and low-risk groups, box plots were used for visualization, and comparisons were made using the Wilcoxon rank-sum test. In addition, stratified survival analysis was conducted, and the KM survival curves were plotted. Finally, the samples were categorized into four groups based on the combination of risk and TMB levels. Stratified survival analysis was performed and KM survival curves were constructed to assess the predictive performance of the model.

### Cell subtyping based on scRNA-seq data

ScRNA-seq data for PCa were obtained from the literature^[13]^. Based on the reported cell labels^[13]^, 835 PCa cells were extracted for downstream analysis. The R package Seurat^[34]^, which is widely used for the systematic processing of scRNA-seq data, was applied for standard data processing. ScRNA-seq data and cell subpopulation annotations were directly adopted from published literature. Downstream analyses were exclusively performed on the PCa cell subpopulations already annotated in these source publications. Quality control standards and parameters were strictly followed according to the processing methods of the original datasets; no additional thresholds were set or adjusted in this study. Gene feature selection was performed to identify highly variable genes using the FindVariable Feature function. PCA^[35]^ was employed to extract the highly variable genes. The optimal number of principal components was determined using the JackStraw and Elbow methods^[34]^. Unsupervised clustering of cells was performed using the FindClusters function with the resolution set to 0.05. The visualization of the clustering results was achieved using the uniform manifold approximation and projection (UMAP) for dimension reduction method^[36]^. In each cell cluster, genes exhibiting differential expression were identified using the FindAllMarkers function at corrected *P*-values of < 0.05 and an absolute log2FC exceeding 1. These distinctive genes with varying expression levels in each cell cluster are presented using both violin plots and heatmaps.

### Identification of high-risk cell subgroups

To identify the high-risk cell subgroups, risk scores were assigned to each cell type using this model. Due to the sparsity of scRNA-seq data, the average risk score was calculated for each cell subgroup based on cell clustering labels. The high-risk cell subgroups were defined as those with the highest risk scores. The risk score results were visually presented using the UMAP^[36]^ and t-distributed stochastic neighbor embedding (t-SNE)^[37]^ dimensionality reduction techniques for enhanced clarity.

We conducted gene set enrichment analysis (GSEA) of the differentially expressed genes associated with these subgroups to explore their biological characteristics. GSEA is frequently used to evaluate changes in pathways and biological processes within expression datasets^[38]^. We used the clusterProfiler^[27,28]^ R package to conduct and visualize GSEA, using a significance threshold with corrected *P*-values of < 0.05.

### Validation of prognostic prediction models at the single-cell level

To validate the prognostic prediction models at a single-cell level, deconvolution was performed on samples from the TCGA-PRAD to assess the proportion of cancer cell subtypes in each sample. The R package CIBERSORT^[15]^, which uses support vector regression, was used to infer the composition of various cell subtypes in the tissue samples. A gene expression profile was constructed for CIBERSORT using differentially expressed genes from each cell subpopulation, which reflected the biological characteristics of each cell subpopulation, thereby maximizing the reliability and accuracy of the results.

Subsequently, all samples were automatically categorized based on the percentage of high-risk cell subpopulations within each sample using the Surv cutoff function. This method separates samples into groups reflecting different levels of high-risk cell subpopulations, with higher levels indicating an increased risk. This grouping is representative of the definition of risk at the single-cell level. Stratified survival analysis was conducted on high- and low-risk cell subgroup samples and the KM survival curves were plotted to validate the model at the single-cell level. Moreover, the ROC curves were generated to assess the predictive accuracy of high-risk cell subgroup composition for 1- and 5-year survival rates.

### Drug resistance analysis

To describe differences in drug resistance, especially that associated with the current first-line treatments, among various cell subtypes, we used the R package oncoPredict^[39]^, applied to the Genomics of Drug Sensitivity in Cancer (GDSC) database^[40]^. Significant differences in drug resistance among subgroups were detected, and combined box plots and violin plots were used for visualization. Statistical significance was determined using the Wilcoxon rank-sum test. The results were presented as a bubble plot.

### Cell culture

Human prostate hyperplasia cell line BPH-1, PCa cell lines PC-3 and 22Rv1 were purchased from American Type Culture Collection (ATCC). All cells were cultured in RPMI-1640 medium supplemented with 10% fetal bovine serum. The cell lines were maintained at 37 °C in a 5% CO_2_ environment. Furthermore, these cell lines were cultured for no more than 20 generations and subjected to routine testing to confirm their absence of mycoplasma contamination.

### Real-time quantitative polymerase chain reaction

Total RNA extraction was extracted using the RNAsimple Total RNA Kit (TIANGEN). The extracted total RNA was dissolved in RNase-free water. cDNA synthesis was performed using the RevertAid First Strand cDNA Synthesis Kit (ThermoFisher) and stored at -20 °C. For quantitative PCR, cDNA was used with SuperReal PreMix Plus SYBR Green Supermix (TIANGEN) in the LightCycler 480 Real-Time PCR System (Roche) following the manufacturer’s instructions. Fluorescence signals were recorded, and β-actin was used as the reference gene. Relative expression levels were analyzed using the 2^-ΔΔCt^ method. The primer sequences are provided in Supplementary Table 1.

### Western blot

Cells were collected, lysed using a lysis buffer, and then centrifuged to collect the supernatant. The supernatant was heated to 95 °C for 5 min. Protein samples were separated by SDS-PAGE electrophoresis and semi-dry transferred onto an NC membrane (Millipore). The membrane was blocked in Tris-buffered saline with 20% Tween-20 containing 5% skim milk for 30 min. Subsequently, the membrane was incubated with the primary antibody at room temperature for 10 min, and then overnight at 4 °C. After undergoing 5 washes, the membrane was incubated with goat anti-mouse/rabbit secondary antibody (Beijing TDY Biotech, 1:10000 dilution) for 40 min, followed by exposure using western ECL Substrate (Millipore). The relative expression levels of each protein were assessed using ImageJ software. The primary antibodies used in this study are listed in Supplementary Table 2. The raw data of blot images for Western blot (WB) analysis are shown in the Supplementary Materials.

### Statistical analysis

All statistical analyses were performed using the GraphPad Prism (version 6.0) or R software (https://www.r-project.org/, version 4.1.2), and presented as mean ± SD. To compare non-normally distributed continuous variables between two groups, we used the Wilcoxon rank-sum test. In cases where not otherwise stated, correlation analyses were conducted using the Spearman method within the “cor” function of the R base package. All *P*-values were two-sided, and the significance threshold, unless otherwise stated, was set at *P-*values of < 0.05.

## RESULTS

### Clustering of tissue samples based on immune features

**Figure 1:**
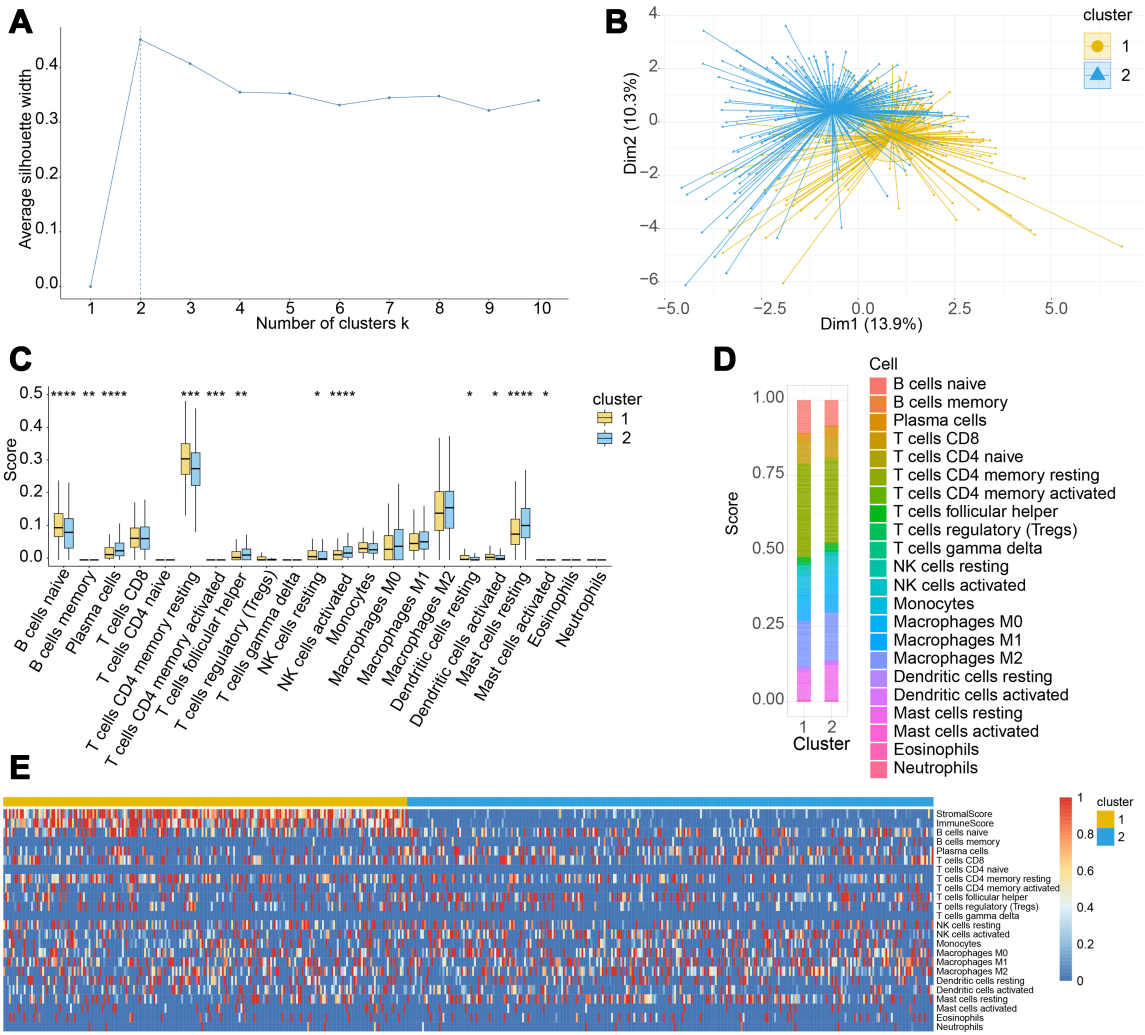
TCGA tissue sample clustering. (A) Optimal cluster number selection. The optimal number is determined by calculating the average silhouette value, with the optimal number corresponding to the maximum silhouette value (dotted line); (B) Dimensionality reduction plot resulting from k-means clustering. Each point on the graph signifies an individual sample and each color represents a sample subgroup; (C) Box plot of immune cell infiltration level. ^*^P < 0.05, ^**^P < 0.01, ^***^P < 0.001, ^****^P < 0.0001, and no symbol indicates no significant difference; (D) Stacked bar chart of the proportions of 22 immune cell infiltrations. The format displayed on the X- and Y-axes illustrates sample groups and the proportion of infiltration levels, respectively; (E) Heatmap of the immune infiltration. The colors represent the infiltration levels or scores. TCGA: The Cancer Genome Atlas.

Evaluation using the fviz_nbclust function with the average silhouette width metric determined k = 2 as the optimal cluster number [Figure 1A]. Subsequent unsupervised k-means clustering partitioned samples into two distinct groups, revealing well-separated clusters in the dimensionality-reduced space [Figure 1B].

In the reduced dimensional space, a clear and distinct boundary was observed between the two sample groups, indicating a robust clustering effect. We examined the differences in the infiltration levels of the 22 immune cell types between the two sample groups using a boxplot [Figure 1C]. Twelve of the 22 immune cell types exhibited significant differences in infiltration levels between the two groups (*P* < 0.05), highlighting variations in the immune characteristics between the groups.

Furthermore, by examining the proportions of different immune cells in the two groups [Figure 1D], we observed variations in immune cell composition. In cluster 1, the proportion of infiltrating naïve B cells was higher than that in cluster 2, which is consistent with the results depicted in the boxplot. A heatmap depicting the infiltration levels of the 22 immune cell types and immune/stromal scores [Figure 1E] revealed significant differences in both the immune and stromal scores between the two clusters. Cluster 1 exhibited higher scores, suggesting a potentially more complex immune microenvironment and immune cell activity within this cluster. These findings collectively reflect the substantial heterogeneity of the immune microenvironment in PCa.

### Biological feature analysis of inter-cluster tissue samples

**Figure 2:**
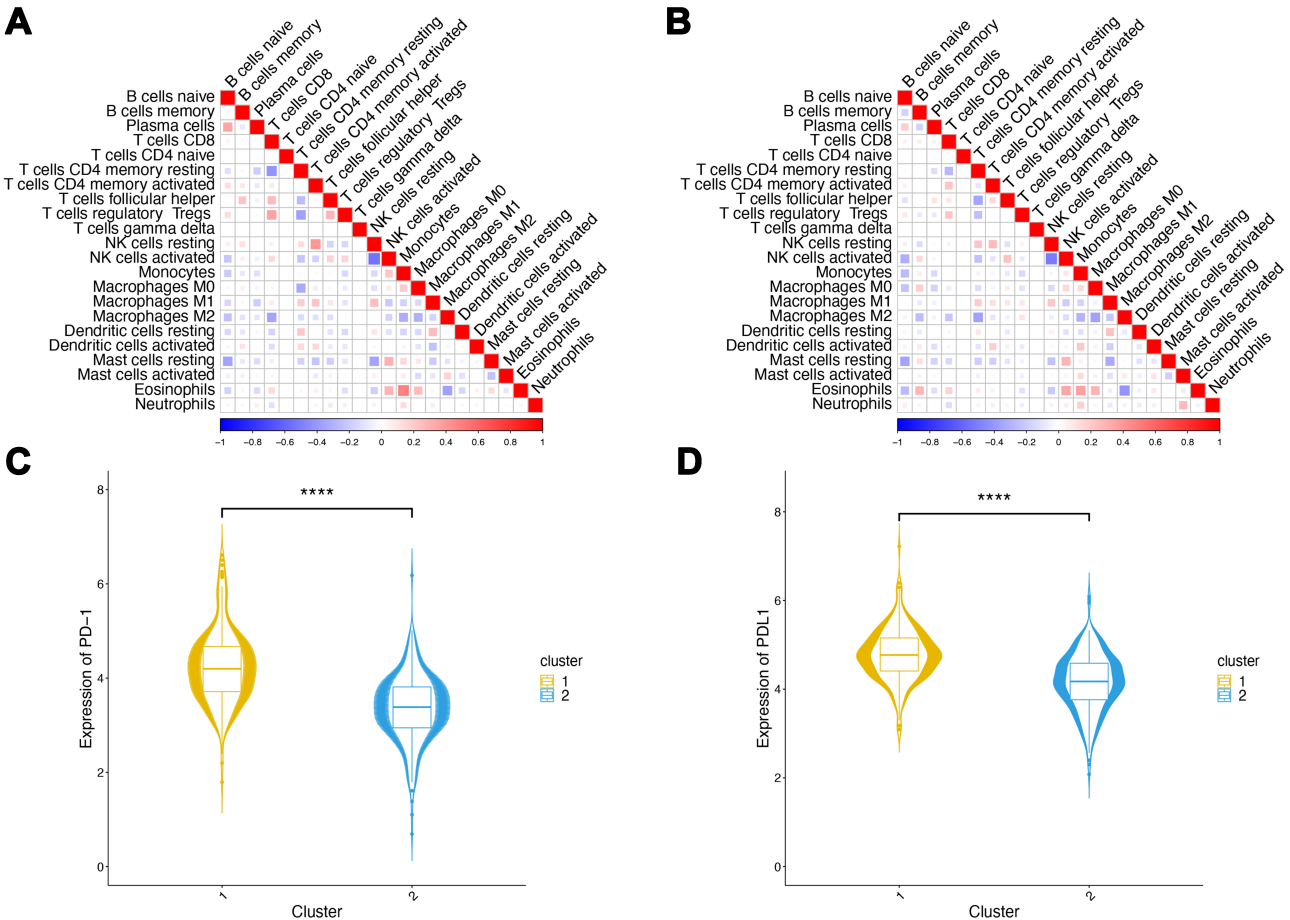
Inter-group immune feature analysis in TCGA. (A) Immune cell correlations in cluster 1. Colors and square sizes in the graph represent the correlations. The larger the square area, the larger the absolute correlation value; (B) Immune cell correlations in cluster 2; (C) Combined plot of *PD-1* gene expression. The symbols above indicate significance levels, with ^****^ denoting *P* < 0.0001; (D) Combined plot of *PD-L1* gene expression. The symbols above indicate significance levels, with ^****^ denoting *P* < 0.0001. TCGA: The Cancer Genome Atlas.

To further analyze the biological differences between clusters 1 and 2, we first examined the correlations between the 22 immune cell types within cluster 1 [Figure 2A] and cluster 2 [Figure 2B]. The results showed differences between immune cell types in the two clusters, with variations primarily observed in the strength of the correlations rather than their directions. Notably, cluster 1 exhibited significantly enhanced positive correlations between plasma cells and naïve B cells, and between eosinophils and monocytes. This intensified co-regulation suggests heightened cooperative activity within the immunologically active microenvironment, including accelerated B cell differentiation from naïve to plasma cell states, and strengthened coordination among innate immune components. Such cellular synergy likely potentiates antitumor immune responses, ultimately influencing disease progression and clinical outcomes. These findings underscore how immune cell interactions drive PCa microenvironmental heterogeneity and modulate immunological activity.

We examined the expression levels of PD-1 and PD-L1 in the two clusters [Figure 2C and D]. Both PD-1 and PD-L1 exhibited highly significant differences in expression levels between the two clusters (*P* < 0.0001), with a higher expression in cluster 1. This observation aligns with our earlier analysis (described in Section “Clustering of tissue samples based on immune features”), where cluster 1 demonstrated higher immune and stromal scores, indicating potentially more immune activity within this cluster.

**Figure 3:**
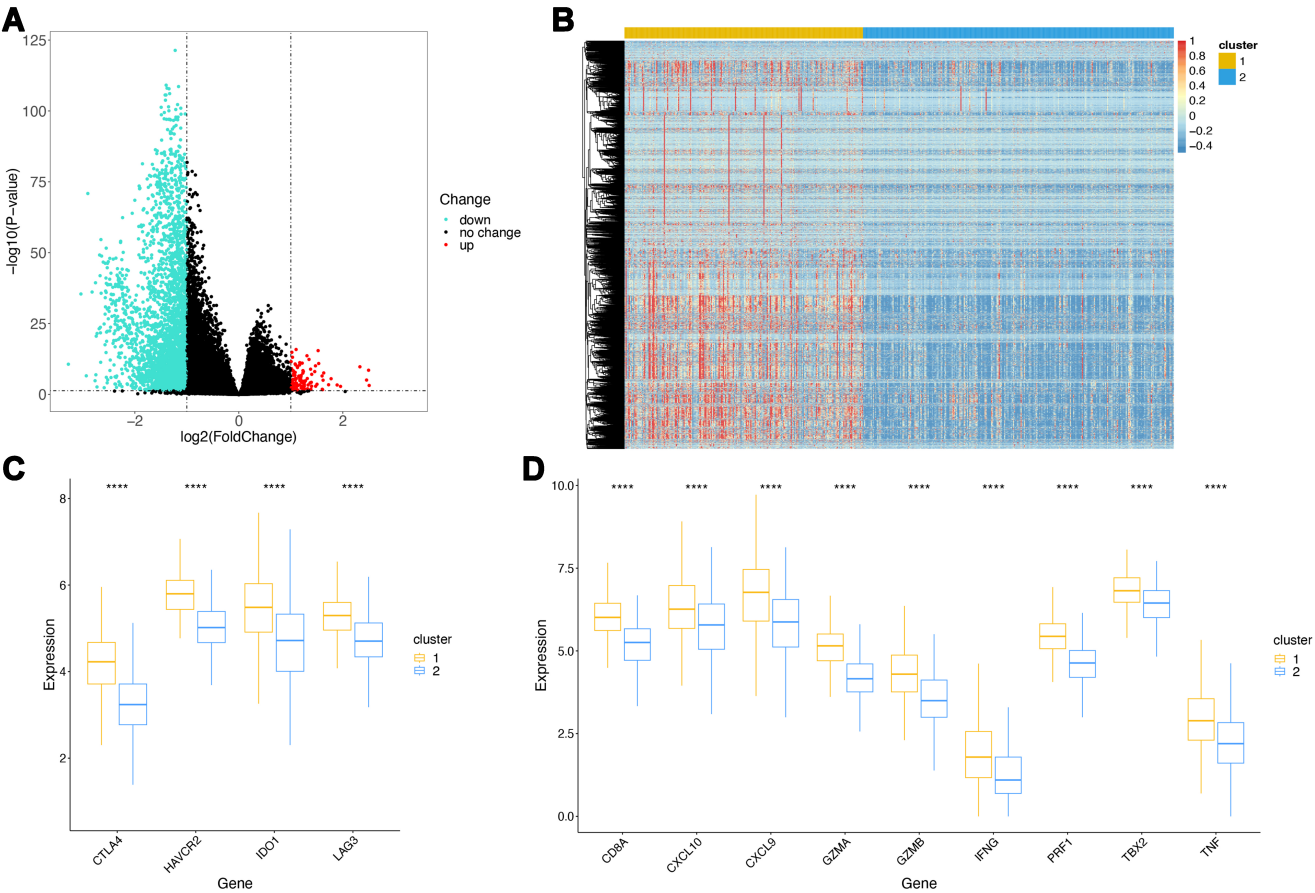
Analysis of differentially expressed genes in TCGA. (A) Volcano plot of differentially expressed genes. Green indicates downregulated genes, red indicates upregulated genes, and black indicates genes with no significant expression changes; (B) Heatmap of differentially expressed genes; (C) Box plots of immune checkpoint-related marker genes. ^****^P < 0.0001. Colors represent sample groups; (D) Box plots of immune activity-related marker genes. ^****^P < 0.0001. Colors represent sample groups. TCGA: The Cancer Genome Atlas.

Subsequently, we conducted differential gene expression analysis between the two clusters, using cluster 1 as the control group. After statistical screening, a total of 3,389 significantly differentially expressed genes were identified, including 102 upregulated and 3,287 downregulated genes [Figure 3A, Supplementary Table 3]. The expression heatmap [Figure 3B] revealed that most genes had significantly higher expression levels in cluster 1, with only a few genes showing higher expression levels in cluster 2. Some immune-related genes have been categorized as immune checkpoint and immune activity-related markers in previous studies. To elucidate the differences in the immune features between the two groups, we determined the expression levels of these genes using boxplots. The results indicated that both immune checkpoint-related marker genes [Figure 3C] and immune activity-related marker genes [Figure 3D] exhibited highly significant differential expression between the two clusters (*P* < 0.0001). Notably, these genes displayed consistently high expression levels in cluster 1, reinforcing the consistency of our findings with previous conclusions.

### Identification of immune-related gene modules using WGCNA

Considering that cluster 1 exhibited complex and active immune characteristics, we further explored the gene modules and identified immune-related gene modules associated with it. To achieve this, we employed the WGCNA method, starting with the identification of gene modules based on significant differential expression between the sample groups, followed by trait association analysis.

**Figure 4:**
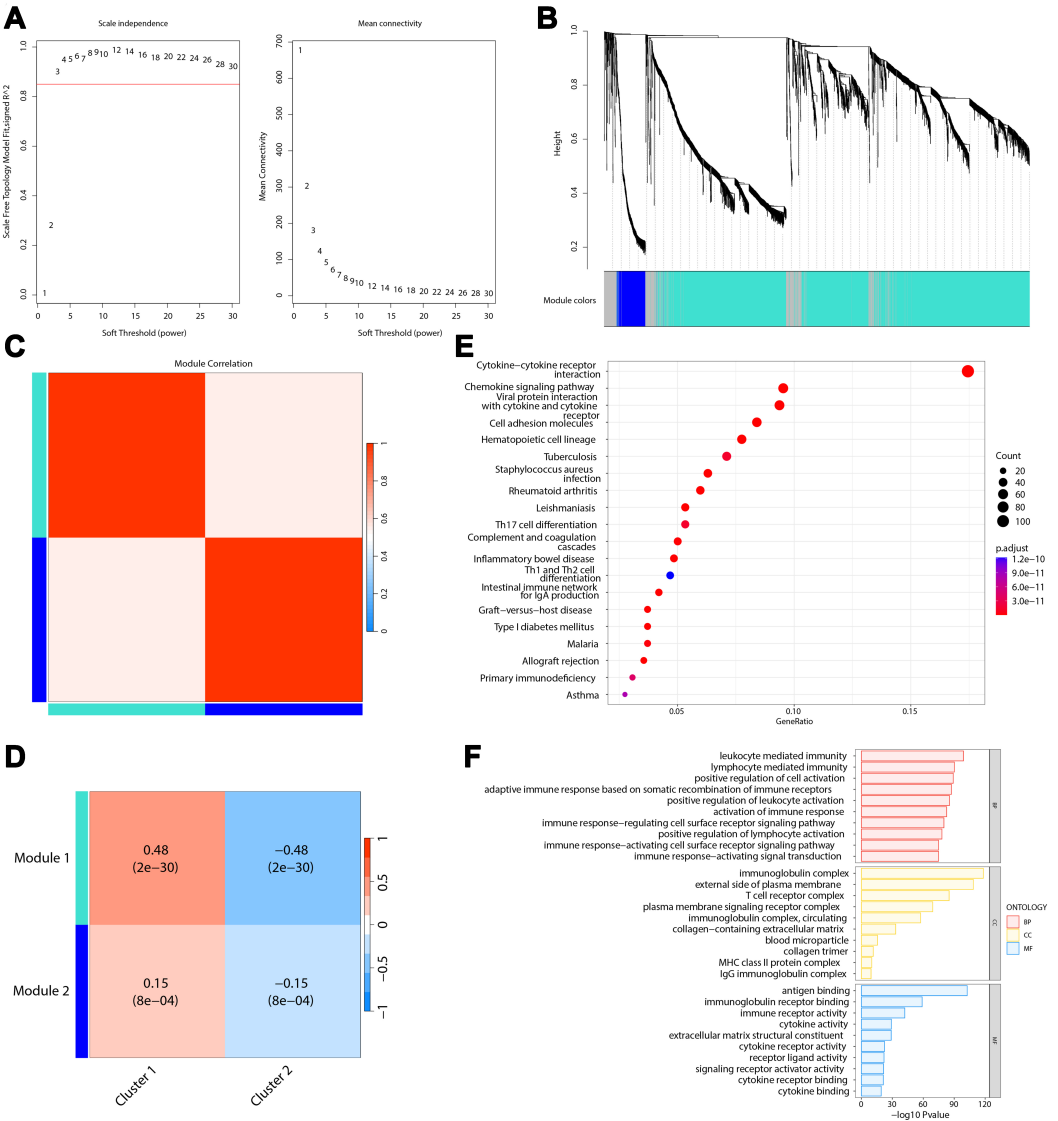
TCGA gene module analysis. (A) Soft-threshold selection. The left plot shows the optimal soft threshold as the first value exceeding the red line, and the right plot typically shows the optimal soft threshold at the point of inflection. The best soft threshold chosen here is 3; (B) WGCNA hierarchical clustering. The top part shows the hierarchical clustering tree, and the bottom part shows the modules corresponding to genes, with each color representing a different module, and the gray module representing an ineffective module; (C) Heatmap of inter-module gene correlations; (D) Heatmap of associations between WGCNA gene modules and sample group labels, displaying the correlations and *P*-values in parentheses; (E) Bubble plot of KEGG enrichment results. The color indicates the corrected *P*-value, with smaller *P*-values closer to red, signifying greater significance; (F) Bar plots of GO enrichment results. TCGA: The Cancer Genome Atlas; WGCNA: weighted gene co-expression network analysis; EKGG: Kyoto Encyclopedia of Genes and Genomes; GO: Gene Ontology.

First, we evaluated the best soft threshold for the WGCNA through computation, and the optimal threshold was determined to be three [Figure 4A]. Using a stepwise network construction approach, we identified two gene modules [Figure 4B] comprising 2,742 and 219 genes [Supplementary Table 4]. The invalid module (gray part in Figure 4B) contained fewer genes, indicating a successful gene module identification process. The correlation analysis between the two gene modules [Figure 4C] revealed a weak positive correlation, signifying good independence between the gene modules. Trait association analysis [Figure 4D] showed that gene module 1 (light blue module in Figure 4B, comprising 2,742 genes) exhibited a stronger association with cluster 1, whereas gene module 2 displayed a weaker correlation. Thus, module 1 may be a key gene module associated with immune characteristics.

To elucidate the biological functions influenced by the 2,742 genes contained within this gene module, we conducted the GO and KEGG enrichment analyses. The KEGG enrichment analysis [Figure 4E, Supplementary Table 5] revealed that the top five significantly enriched pathways were cytokine-cytokine receptor interactions, viral protein interactions with cytokines and cytokine receptors, hematopoietic cell lineages, chemokine signaling pathways, and cell adhesion molecules. These pathways are primarily associated with cytokine and chemokine activity.

The GO enrichment analysis [Figure 4F, Supplementary Table 6] showed that the top five significantly enriched terms were the immunoglobulin complex, external side of the plasma membrane, antigen binding, leukocyte-mediated immunity, and lymphocyte-mediated immunity. These results were predominantly related to the immune processes and were largely aligned with the KEGG enrichment results.

### Construction and validation of prognostic prediction model

To reveal a clinically relevant relationship between immune features and outcomes, we constructed a prognostic prediction model based on the genes contained in gene module 1.

**Figure 5:**
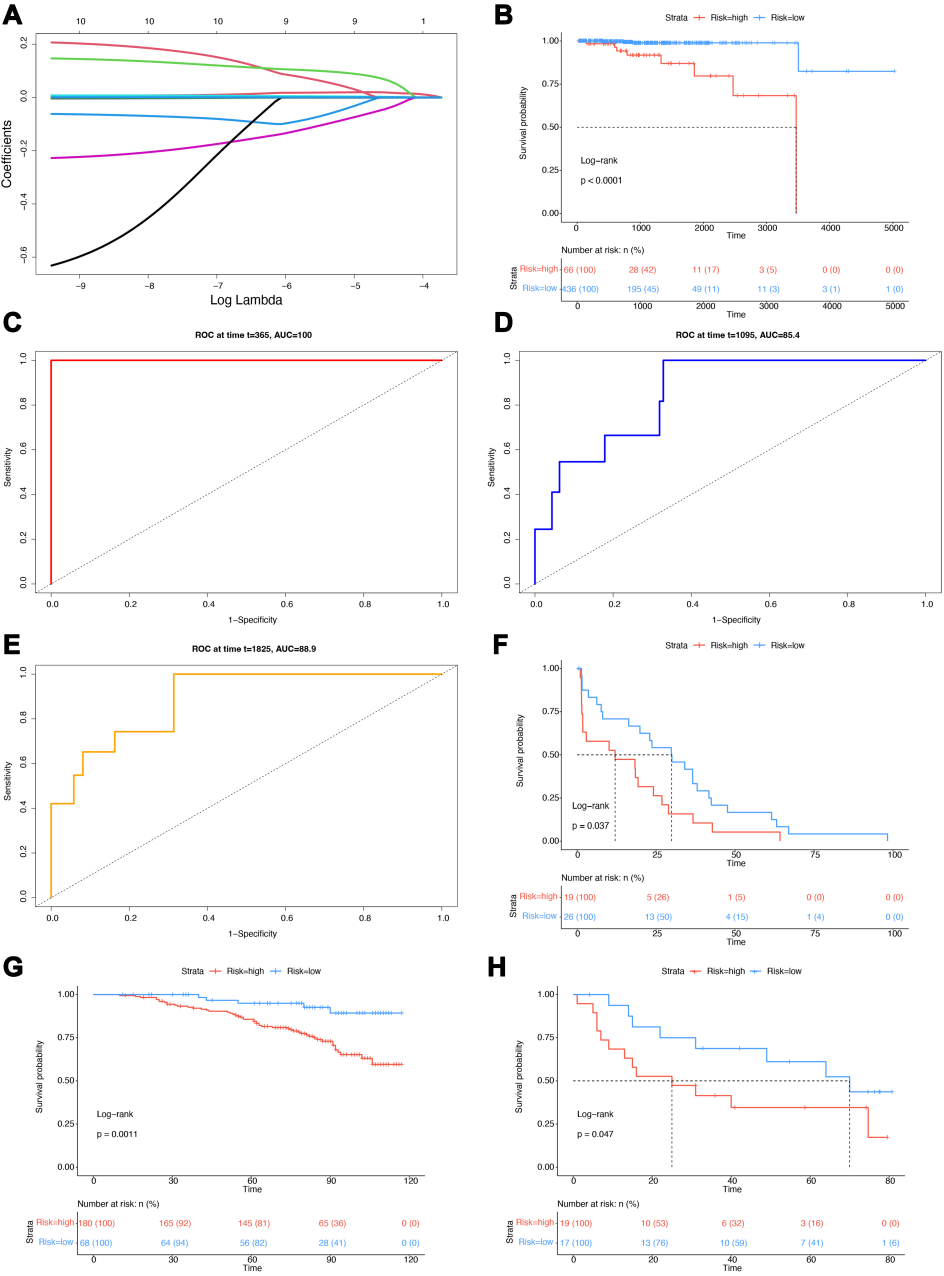
TCGA prognostic prediction model and validation. (A) LASSO regression curve; (B) Survival curves for high- and low-risk groups. The top part shows the survival curves. The dashed line corresponds to the median survival time. The bottom part shows the risk table. The colors match the survival curves, and the table displays data on the number of surviving samples and their percentage of the total group; (C) ROC curve for 1-year survival rate prediction; (D) ROC curve for 3-year survival rate prediction; (E) ROC curve for 5-year survival rate prediction; (F) Survival curves for the validation datasets GSE70769; (G) Survival curves for the validation datasets GSE116918; (H) Survival curves for the validation datasets GSE4660. TCGA: The Cancer Genome Atlas; LASSO: least absolute shrinkage and selection operator; ROC: receiver operating characteristic.

Using survival data from the TCGA-PRAD samples, we conducted a univariate Cox regression analysis to screen for genes significantly associated with prognosis. This process identified 10 genes: *CD22*, *RAB33A*, *CCDC3*, *GYPE*, *TREM1*, *NFE2*, *CEP295NL*, *HORMAD1*, *VSTM1*, and *MUC5B*. The LASSO regression analysis [Figure 5A] results showed that the coefficients of these 10 genes in the regression model were all non-zero; hence, all were retained. The final model was as follows:

*Score* = -0.0016 × ExpCD227 + 0.0106 × ExpRAB33A7 - 0.0008 × ExpCCDC37 - 0.08257 × ExpGYPE7 + 0.0063 × ExpTREM17 - 0.1717 × ExpNFE27 - 0.19027 × ExpCEP295NL7 + 0.1462 × ExpHORMAD17 + 0.1186 × ExpVSTM17 + 0.00057 × ExpMUC5B7.

Based on the model, a risk score was assigned to the samples, and all samples were subsequently divided into high- and low-risk groups. The KM survival curves showed a significant difference in prognosis between the two groups (log-rank test *P* < 0.0001; Figure 5B). To validate the predictive performance of the model, we plotted the ROC curves to predict 1-, 3-, and 5-year survival rates. The results demonstrated that the model achieved an AUC of 1 for 1-year survival prediction [Figure 5C], 0.854 for 3-year survival [Figure 5D], and 0.889 for 5-year survival [Figure 5E].

To further validate the generalizability and robustness of the model, three external datasets, namely GSE46602, GSE70769, and GSE116918, were used. The model was applied to each dataset for risk scoring, survival analysis, and the KM curve plotting. The results showed significant differences in the survival time between the high- and low-risk groups in the GSE70769 [Figure 5F], GSE116918 [Figure 5G], and GSE46602 [Figure 5H] datasets, with log-rank test *P*-values of 0.037, 0.0011, and 0.047, respectively. These findings provide strong evidence that the model exhibited good generalizability, stability, and robustness.

### Biological feature analysis between high- and low-risk groups

**Figure 6:**
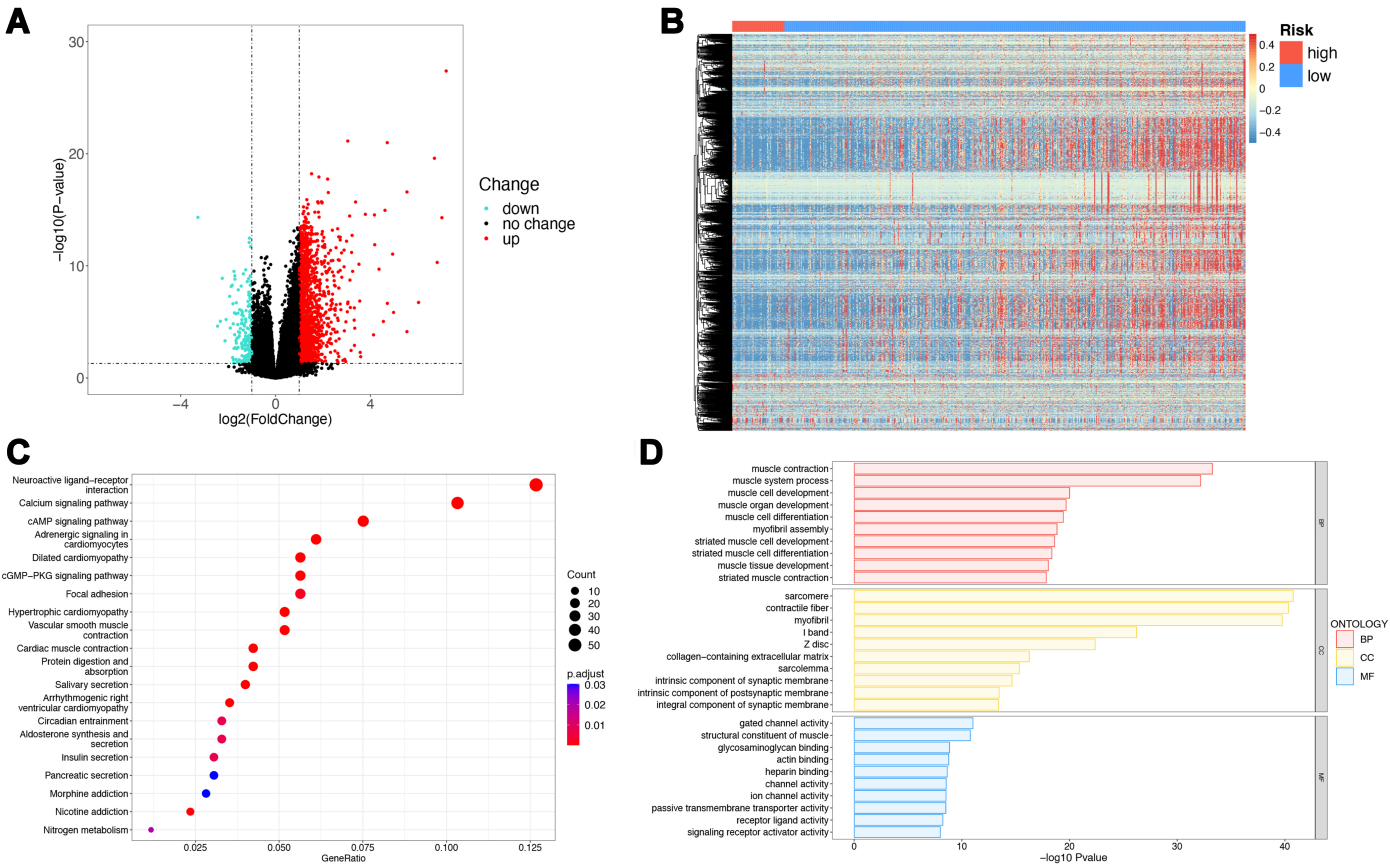
Transcriptome analysis of high and low-risk groups in TCGA. (A) Volcano plot of differentially expressed genes. Green indicates downregulated genes, red indicates upregulated genes, and black indicates genes with no significant expression changes; (B) Heatmap of differentially expressed genes; (C) Bubble plot of KEGG enrichment results. The X-axis represents the gene ratio. The Y-axis represents pathway names, with the size of the points representing the number of genes enriched in that pathway. The color indicates the corrected *P*-value, with smaller *P*-values closer to red, signifying greater significance. Only the top 20 pathways are shown here; (D) Bar plots of GO enrichment results. Only the top 10 most significant GO terms for BP, CC, and MF are shown. TCGA: The Cancer Genome Atlas; KEGG: Kyoto Encyclopedia of Genes and Genomes; GO: Gene Ontology; BP: biological process; CC: cell component; MF: molecular function.

At the transcriptome level, differential gene expression analysis was performed to compare the groups, with the high-risk group used as the control. After statistical screening, 1,796 genes demonstrated significant differential expression [Supplementary Table 7], with 1,587 genes upregulated and 209 genes downregulated [Figure 6A]. The expression heatmap [Figure 6B] indicated that the most significantly differentially expressed genes exhibited higher expression in the comparative group, with only a small number showing higher expression in the high-risk group. The GO and KEGG enrichment analyses were performed to elucidate biological functions. The KEGG enrichment analysis results [Figure 6C, Supplementary Table 8] showed that the top five significantly enriched pathways were primarily linked to muscle cells, including the calcium signaling pathway, neuroactive ligand-receptor interaction, dilated cardiomyopathy, hypertrophic cardiomyopathy, and adrenergic signaling in cardiomyocytes. The GO enrichment analysis results [Figure 6D, Supplementary Table 9] indicated that the top five significantly enriched GO terms were related to muscle systems, including sarcomere, contractile fiber, myofibril, muscle contraction, and muscle system processes. These findings suggest that the primary biological distinctions between the two groups are related to muscle cells or the muscular system.

**Figure 7:**
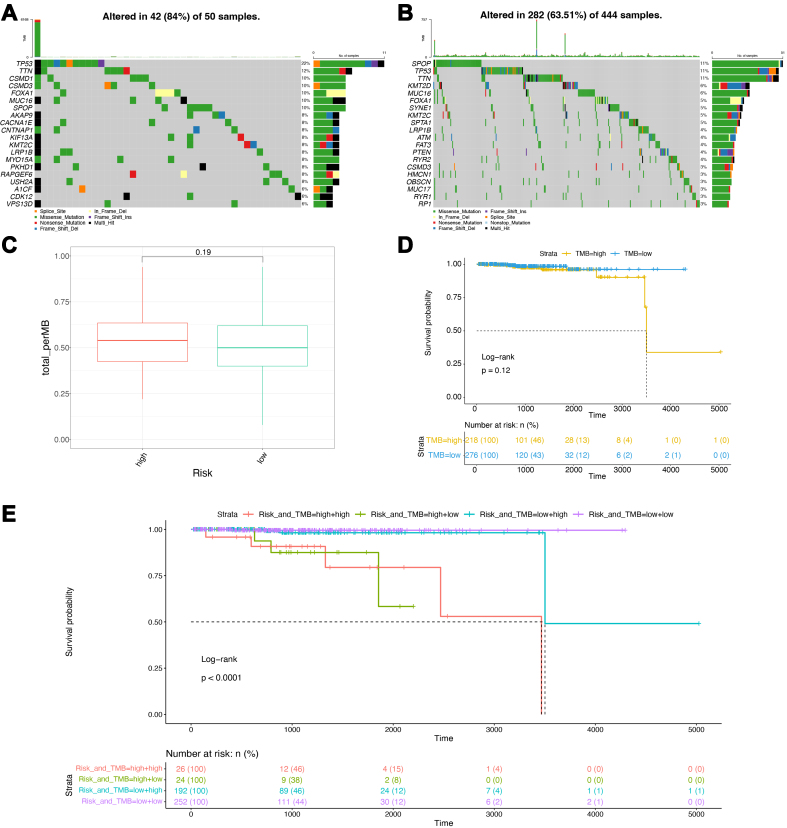
Genomic analysis of high and low-risk groups in TCGA. (A) Waterfall plot of gene mutations in the high-risk group; (B) Waterfall plot of gene mutations in the low-risk group; (C) Box plots of TMB scores; (D) Survival curves for the high and low TMB groups; (E) Survival curves for the combined groups of model scores and TMB. TCGA: The Cancer Genome Atlas; TMB: tumor mutation burden.

The analysis of genomic mutations in the high- and low-risk groups showed that the former group exhibited a higher gene mutation rate [Figure 7A], which was characterized by TP53 mutations, whereas SPOP mutations were predominant in the low-risk group [Figure 7B]. These findings reflected a higher level of malignancy in the high-risk group. The TMB is a crucial indicator of the effectiveness of immunotherapy. Therefore, we compared the TMB levels between the two groups [Figure 7C]. The results showed that the high-risk group had a slightly higher TMB, although this difference was not statistically significant (*P* = 0.19). Further analysis was conducted to determine any differences in the prognosis. Using TMB levels, all samples were reclassified into high- and low-TMB groups. The KM survival curves suggested that the low-TMB group exhibited a more favorable prognosis than the high-TMB group [Figure 7D]; however, this difference was not statistically significant (*P* = 0.12), indicating that TMB alone cannot serve as an independent predictor of prognosis in patients with PCa. To underscore the effectiveness of the prognostic prediction model constructed in this study, a combined analysis was performed, incorporating model scores (i.e., high- and low-risk groups) and TMB levels (i.e., high- and low-TMB groups). Four-stratified KM survival curves were plotted [Figure 7E], and the results showed a significant overall difference in the prognosis (*P* < 0.0001). The group with the best prognosis was the low-risk + low-TMB group, while the group with the worst prognosis was the high-risk + high-TMB group. These results demonstrate the value of the proposed model, which could be combined with the TMB levels to improve PCa prognostication.

### Unsupervised clustering of cancer cell subtypes using scRNA-seq data

**Figure 8:**
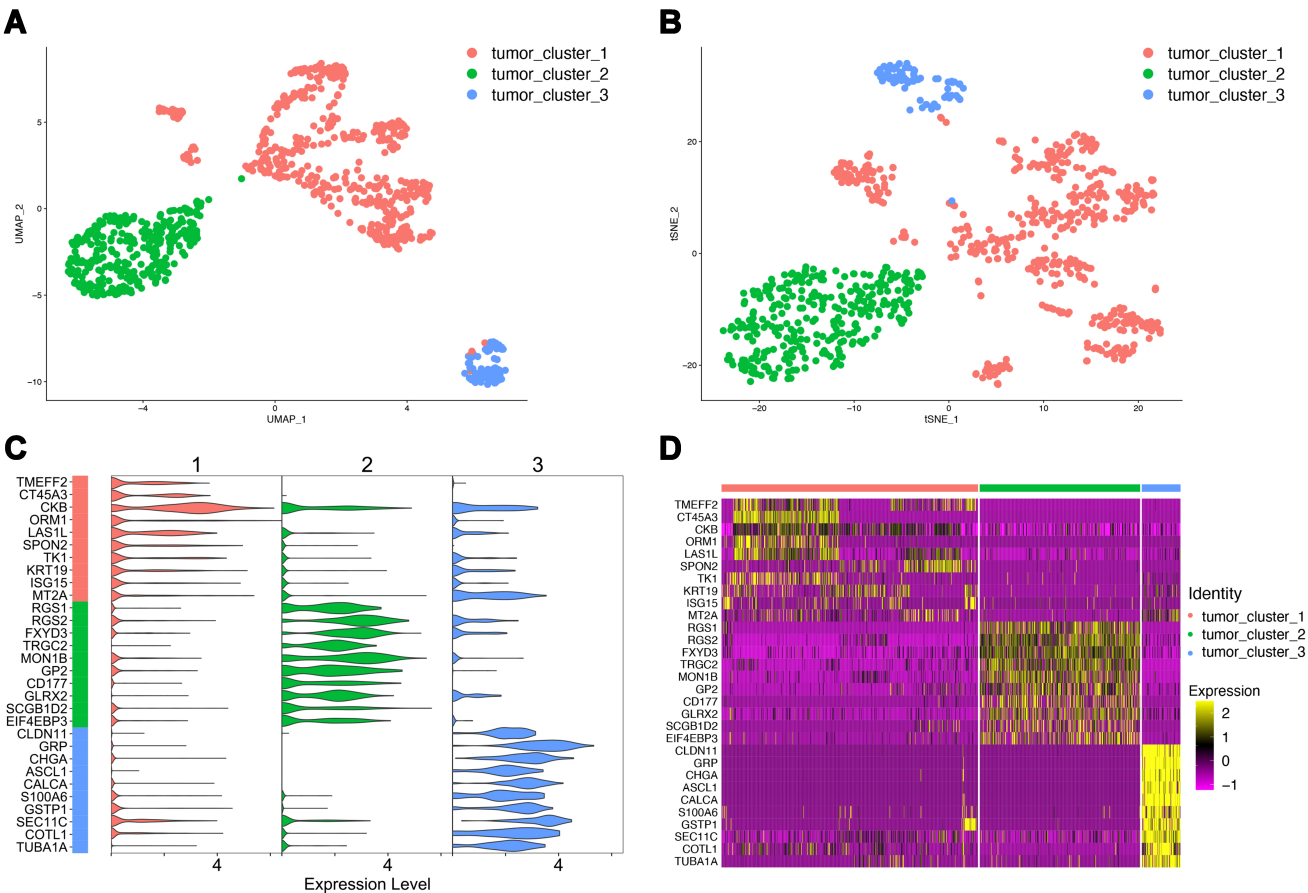
Unsupervised clustering of PCa cells. (A) UMAP dimensionality reduction results for unsupervised cell clustering. Colors represent different cell subgroups; (B) t-SNE dimensionality reduction results for unsupervised cell clustering. Colors represent different cell subgroups; (C) Violin plots of the top 10 significantly differentially expressed genes in each subgroup; (D) Heatmap of the top 10 significantly differentially expressed genes in each subgroup. PCa: Prostate cancer; UMAP: uniform manifold approximation and projection; t-SNE: t-distributed stochastic neighbor embedding.

PCa scRNA-seq expression data were obtained from previous studies. The PCa cell data were extracted from this dataset using known cell labels, resulting in 835 cells. Quality control and dimensionality reduction were performed according to the Seurat analysis workflow. The optimal number of principal components was assessed using the JackStraw and Elbow software. The results indicated that the first 12 principal components had *P*-values of < 0.05 in JackStraw [Supplementary Figure 1A], and principal component 12 was located at the inflection point in the elbow [Supplementary Figure 1B]. Therefore, the first 12 principal components were selected for downstream analysis. Three distinct cancer cell subgroups were identified using unsupervised clustering. These subgroups were visualized using the UMAP [Figure 8A] and tSNE [Figure 8B] methods. Gene differential expression analysis [Figure 8C, Supplementary Table 10] revealed that cancer cell subgroup 1 was characterized by high expression of genes such as TMEFF2, subgroup 2 by high expression of genes such as RGS1, and subgroup 3 by high expression of genes such as CLDN11. The expression heatmap [Figure 8D] demonstrates that the significantly differentially expressed genes within each cancer cell subgroup consistently exhibited high expression within their respective cells, reflecting the intratumor heterogeneity of PCa.

### High-risk PCa cell subtypes and their biological features

**Figure 9:**
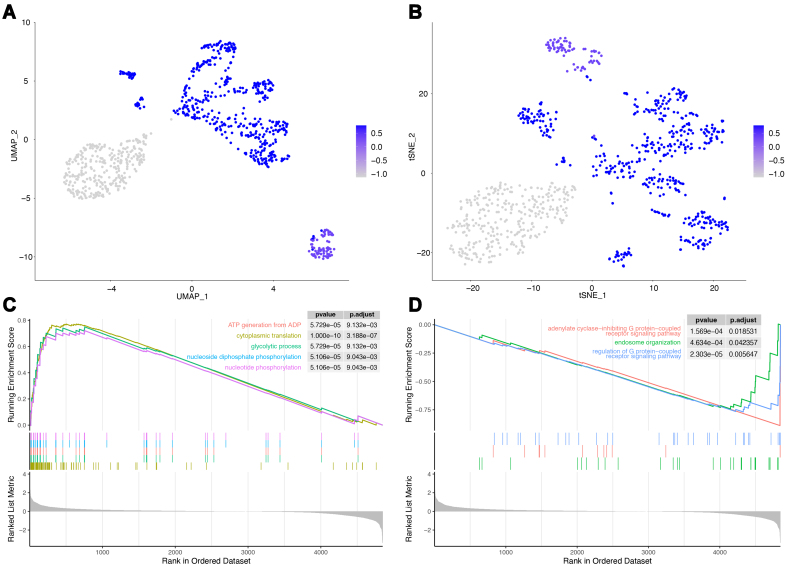
High-risk PCa cell subgroups. (A) Risk score UMAP visualization; (B) Risk score t-SNE visualization; (C) Upregulated GSEA enrichment results. The upper Y-axis represents the enrichment score, and the lower Y-axis represents the log fold change (logfc) values. Only the top 5 functions are shown; (D) Downregulated GSEA enrichment results. PCa: Prostate cancer; UMAP: uniform manifold approximation and projection; t-SNE: t-distributed stochastic neighbor embedding; GSEA: gene set enrichment analysis.

The prognostic model developed in this study was used to score various cancer cell subgroups, enabling the identification of high-risk subgroups among the three cancer cell subtypes. The results of dimensionality reduction using UMAP [Figure 9A] and tSNE [Figure 9B] revealed that the risk scores for the three cancer cell subgroups were ranked from highest to lowest as follows: Subgroups 1, 3, and 2. Therefore, the cancer cell subgroup with the highest risk score, subgroup 1, was defined as the high-risk subgroup for further analysis.

To uncover the distinctive biological characteristics of subgroup 1 cells compared to other cancer cell subgroups, the GSEA was conducted based on the results of the differential expression analysis for subgroup 1. Following statistical filtering, 35 significantly enriched functions were identified, with 32 upregulated and 3 downregulated functions [Supplementary Table 11]. The top five significantly upregulated functions included “cytoplasmic translation”, “glycolytic process”, “ATP generation from ADP”, “nucleoside diphosphate phosphorylation”, and “nucleotide phosphorylation” [Figure 9C]. These functions are primarily associated with energy metabolism, suggesting that the high-risk cancer cell subgroup may exhibit more active energy metabolism. Meanwhile, the downregulated functions consisted of “endosome organization”, “adenylate cyclase-inhibiting G protein-coupled receptor signaling pathway”, and “regulation of G protein-coupled receptor signaling pathway” [Figure 9D], which are primarily related to G protein-coupled receptors. These findings collectively provide insights into the distinctive characteristics of high-risk cancer cell subgroups, particularly those related to energy metabolism, and highlight the potential involvement of G-protein-coupled receptors.

### Validation of the immune-related prognostic prediction model in clinical samples

Based on the assumption that a higher proportion of subgroup 1 cells is associated with a worse prognosis, this study aimed to validate the accuracy and reliability of the proposed prognostic model at the single-cell level.

**Figure 10:**
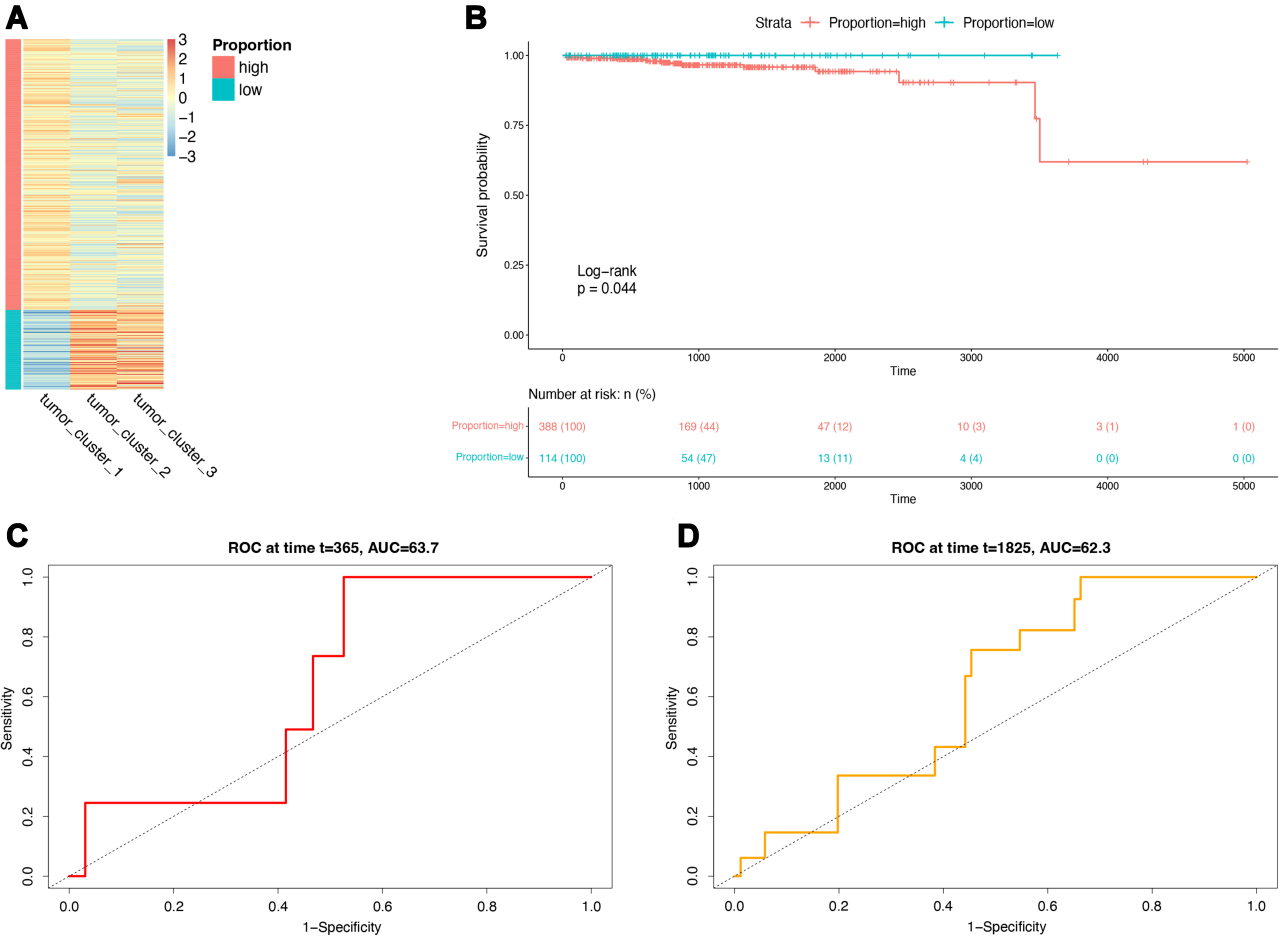
Single-cell level validation of the model. (A) Heatmap of subgroup proportions. Colors represent proportions (scores); (B) Survival curves for high and low-proportion groups; (C) ROC curve for 1-year survival rate prediction; (D) ROC curve for 5-year survival rate prediction. ROC: Receiver operating characteristic.

The CIBERSORT deconvolution algorithm was initially employed to assess the composition of cell subgroups in samples from the TCGA-PRAD. Subsequently, the samples were categorized into two groups, namely, high- and low-content groups of high-risk cell subgroup 1 [Figure 10A]. Survival analysis was conducted for these two groups and the KM survival curves were generated [Figure 10B]. The results indicated a significant difference in prognosis between the high-content and low-content groups (log-rank *P* = 0.044), with the high-content group exhibiting a worse prognosis, which is consistent with our initial hypothesis. In addition, the ROC curves were plotted to predict the 1- and 5-year survival rates, resulting in the AUC values of 0.637 [Figure 10C] and 0.623 [Figure 10D], respectively.

### Analysis of drug resistance at the single-cell level

**Figure 11:**
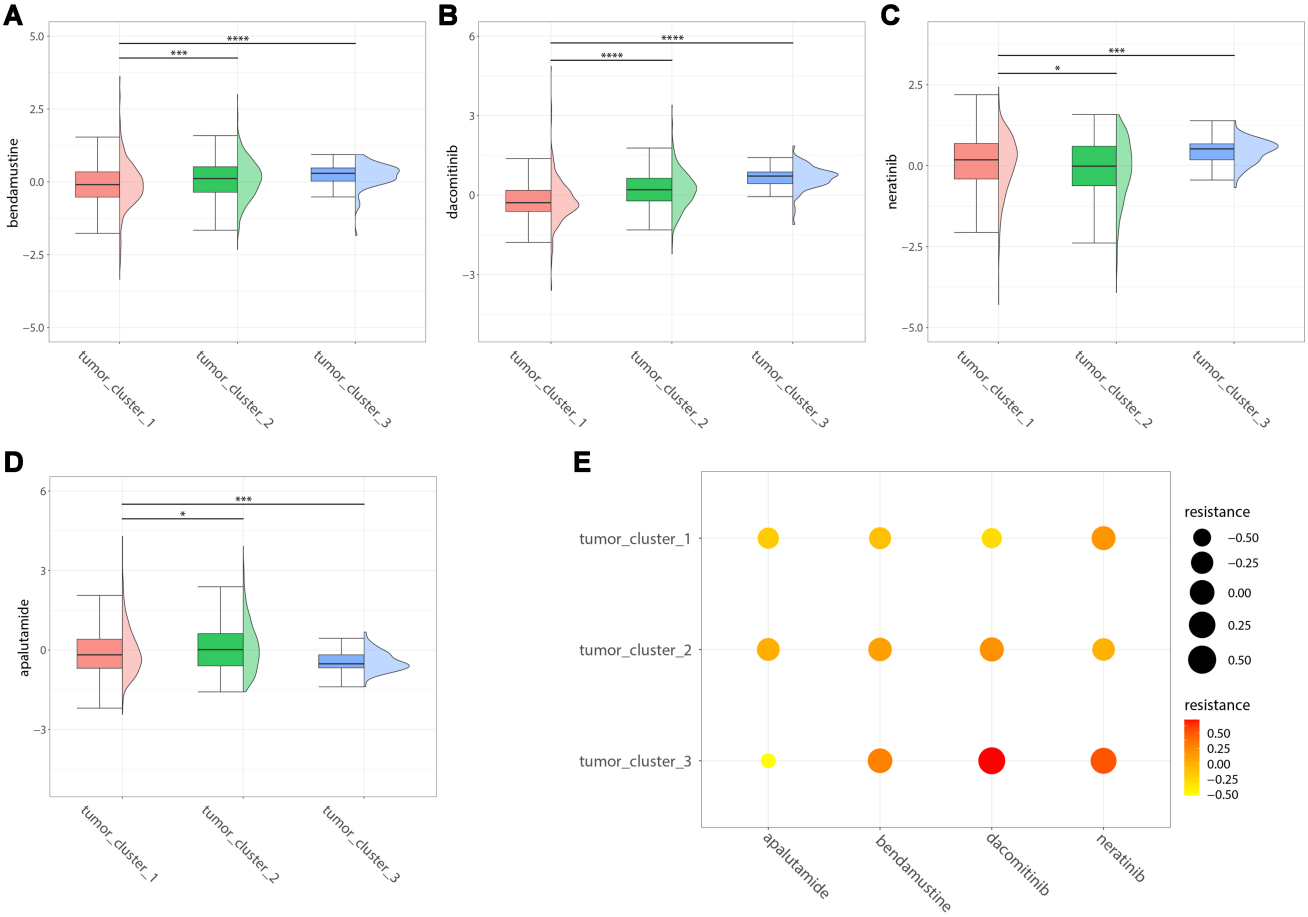
Drug resistance analysis. (A) Composite plot of bendamustine resistance; (B) Composite plot of apalutamide resistance; (C) Composite plot of dacomitinib resistance; (D) Composite plot of neratinib resistance; (E) Resistance bubble chart. ^*^P < 0.05, ^**^P < 0.01, ^***^P < 0.001, and ^****^P < 0.0001.

Integrating drug resistance data obtained from experimental measurements in the GDSC and PCa cell line expression profiles and utilizing the oncoPredict method, we evaluated the resistance to four first-line anticancer drugs within the three cancer cell subgroups. These drugs include bendamustine, apalutamide, dacomitinib, and neratinib. The results revealed significant differences in drug resistance among the various cell subgroups (*P* < 0.05). Specifically, cancer cell subgroup 1 displayed higher sensitivity (lower resistance) to bendamustine [Figure 11A] and dacomitinib [Figure 11B], subgroup 2 was more sensitive to neratinib [Figure 11C], and subgroup 3 exhibited increased sensitivity to apalutamide [Figure 11D]. Overall [Figure 11E], each cell subgroup demonstrated distinct tolerance and sensitivity to the four anticancer drugs. Therefore, the optimal drugs for specific cancer cell subgroups may differ, potentially explaining the variation in patient responses to the same drugs, which may be accounted for by the inherent intratumor heterogeneity.

### Validation of the expression of the model genes

**Figure 12:**
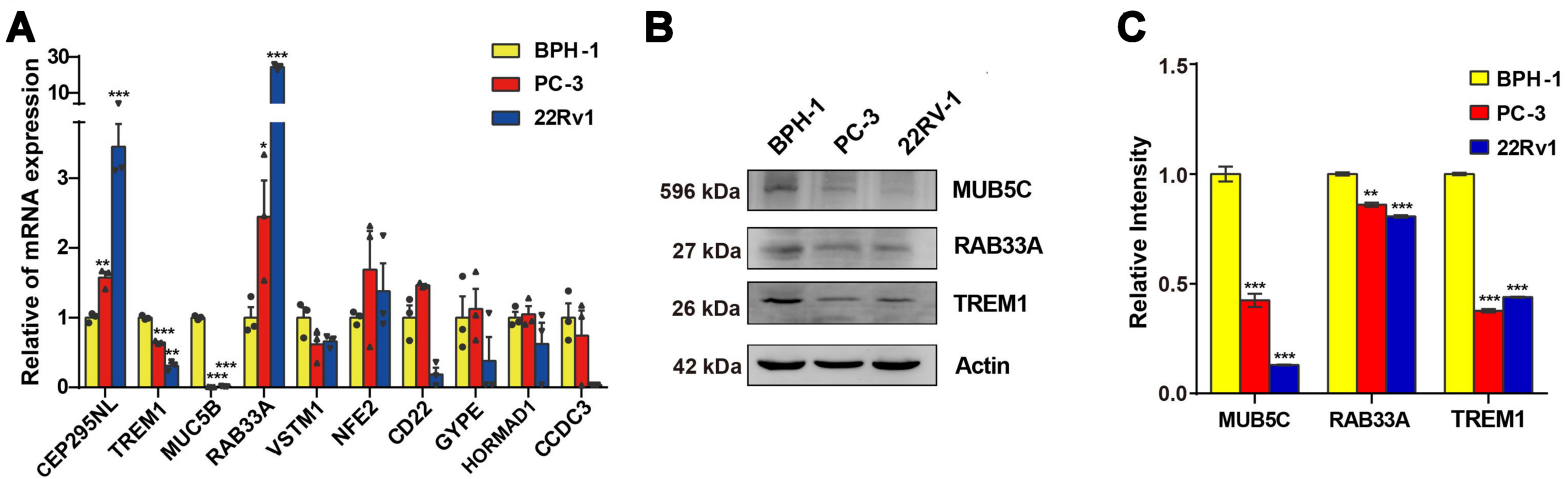
Comparison of PCa cell lines with BPH-1 cells, with 3 biological replicates. (A) qRT-PCR detection of differential expression of 10 genes between different groups; (B) Molecules detected as positive in qRT-PCR, subsequently tested using WB; (C) Quantify the blots from 3 replicates comparing the protein levels between different groups. PCa: Prostate cancer; qRT-PCR: real-time quantitative polymerase chain reaction; WB: Western blot. ^*^P < 0.05, ^**^P < 0.01, ^***^P < 0.001.

The expression validation of the ten model genes was performed on human prostate hyperplasia cells and PCa cells by real-time quantitative polymerase chain reaction (qRT-PCR) and WB analysis. Notably, we observed a significantly higher expression of CEP295NL and RAB33A in PCa cell lines compared to BPH-1 cells. Conversely, TREM1 and MUC5B displayed significantly lower expression in PC-3 and 22Rv1 cells compared to BPH-1 [Figure 12A]. In the WB analysis, MUC5B and TREM1 exhibited significantly higher expression in both PCa cell lines. However, it is worth noting that the expression of RAB33A was notably higher in the normal prostate epithelial cell group compared to both PCa tissue sample groups, which contradicted the qRT-PCR results [Figure 12B and C]. Therefore, MUC5B and TREM1 could be considered reliable and precise model genes for PCa.

## DISCUSSION

PCa affects men’s health worldwide. Currently, the primary treatments for PCa include hormones, radiation, and chemotherapy. However, the prognosis for most patients remains poor and the mortality rate continues to be high^[41]^, creating a need for new treatment strategies and personalized therapies that may improve outcomes. Many studies have shown that dynamic communication between tumor cells and the immune microenvironment accelerates tumor growth and disease progression^[4]^. Understanding the tumor immune microenvironment and the relevant biomarkers may improve the diagnosis and treatment of PCa.

To investigate the PCa immune microenvironment, we employed unsupervised clustering methods to stratify patients from the TCGA-PRAD database into two subgroups with distinct immune features, and we subsequently compared them. Within the immune microenvironment, we observed higher infiltration of cluster B cells, indicating enhanced immune activity in this subgroup. In high-risk PCa, the secretion of chemokine CXCL13 promoted increased B cell infiltration within the tumor, with B lymphocytes playing a role in the development of castration-resistant PCa by generating lymphotoxins^[42]^. Similarly, samples from patients with recurrent or progressive PCa displayed higher and more abundant B cell infiltration^[43]^. Furthermore, the immune score, a critical parameter for studying immune features, exhibited significant variations among the different subgroups, suggesting that patients with PCa and distinct immune characteristics might have varying prognoses and treatment responses.

We investigated interactions among the immune cells within the PCa microenvironment. We analyzed the correlations between 22 different immune cell types within the two subgroups. The observed enhanced correlations between specific immune cell types in cluster 1, particularly plasma cells and naïve B cells, as well as eosinophils and monocytes, suggest intensified intercellular collaboration within the immunologically active TME. This pattern may reflect heightened B cell differentiation and innate immune cell coordination, a process supported by prior studies to facilitate antigen presentation and antitumor immunity. Understanding these correlation dynamics helps unravel PCa immune heterogeneity and provides a scientific rationale for immunomodulatory therapeutic strategies. Furthermore, significant differences were observed in the expression of PD-1 and PD-L1 among the different immune subgroups. Immune checkpoint molecules such as PD-1 and PD-L1 may be involved in tumor immune evasion, and their high expression levels indicate a positive response to immunotherapy^[44]^. In addition, various biomarkers associated with immune activity and immunotherapy responses displayed differential expression, further highlighting the heterogeneous nature of PCa immune characteristics. Therefore, the identification of gene modules related to immune characteristics is crucial for the improved classification of patients with PCa.

In our study, a set of immune-related genes was derived from the WGCNA modules using univariate Cox analysis and was used for prognostication. These genes exhibit a strong correlation with patient prognosis, indicating their potential as clinical tools for risk stratification, particularly CCDC3. Previously, Ke *et al.* employed multivariate Cox analysis to identify immune markers closely associated with PCa prognosis and discovered that high expression of CCDC3 in cancer cells is more likely to lead to radiation resistance^[45]^. The inhibition of CCDC3 expression in PCa cell lines significantly inhibited cancer cell migration and invasion^[45]^. However, in LASSO regression analyses within the TCGA cohort, CCDC3 did not demonstrate a significant association with poor prognosis or drug resistance. We postulate that this may be attributed to differences in cohort composition, analytical methodologies, and biological context. Furthermore, genes in our model such as MUC5B and TREM1 have been documented in relevant literature for their roles in the tumor immune microenvironment and prognosis^[46,47]^, underscoring the model’s multidimensional novelty and clinical applicability. Considering the role of post-transcriptional regulation in mRNA dynamics, the discordant results for RAB33A between qRT-PCR and WB analyses may be attributable to translational suppression. Furthermore, activation of the ubiquitin-proteasome system or autophagy pathways could contribute to the specific degradation of RAB family proteins. Notably, the aberrantly activated degradation pathways that may be present in the PCa microenvironment warrant particular consideration. Collectively, our results reveal the critical value of immune-related genes in PCa prognosis assessment and provide direction for subsequent mechanistic investigations.

Conventional assessment systems, such as TNM staging, often have limitations in accurately predicting patient prognoses. The integration of diverse biomarkers encompassing different types and functions into a predictive model substantially improves the precision of prognostic prediction and offers valuable insights into treatment decisions. Feng *et al.* leveraged data from the TCGA and GEO databases to identify lncRNAs associated with aging. These age-related lncRNAs were subsequently used as potential biomarkers to build a prognostic model for patients with PCa^[48]^. The scoring system used within this model effectively evaluates patient prognosis^[48]^. Herein, we used the LASSO analysis to construct an immune-related predictive model for the prognosis of patients with PCa. The results demonstrated that our model has robust predictive capability for 3- and 5-year survival, with AUC values significantly exceeding 0.85. However, the 1-year survival AUC reached 1.0, a result primarily due to the extremely low number of events (deaths) within the first year in the TCGA-PRAD cohort, leading to inflated performance metrics at this time point. Consequently, the 1-year AUC should be interpreted with caution and not considered a reliable indicator of model performance. The 3- and 5-year AUC values more accurately reflect the model’s true predictive power. Future studies incorporating larger sample sizes and independent prospective cohorts are warranted to further validate the model’s accuracy for short-term prediction. Additionally, the model predicted differences in prognosis between high- and low-risk patients when used alone or in combination with the TMB subgroups in survival analysis, confirming the critical role of immune features in tumor development. Notably, primary PCa typically exhibits low TMB, which likely partially accounts for the lack of statistically significant differences in our cohort. Collectively, these findings suggest that TMB alone has limited clinical utility as a prognostic indicator within the context of primary PCa. To explore the underlying reasons for the differences in prognosis, we analyzed the biological characteristics of the two groups. The high-risk group had a significantly higher gene mutation rate, primarily centered on TP53. TP53 is a well-known tumor suppressor gene that helps maintain genomic stability and cancer resistance^[49]^. Mutations in TP53 result in the loss of its tumor-suppressive function, increased cell resistance, and promotion of PCa progression^[50]^. Moreover, the high gene mutation rate in the high-risk group can generate more neoantigens, which enhance antigen presentation and lead to increased active immune activity within the TME.

The prognostic model we established not only stratifies patients into high- and low-risk groups, but also reveals subtype-specific drug tolerance patterns. Notably, the 10-gene signature demonstrates strong predictive value for both prognosis and therapeutic response. For instance, high-risk patients identified by the model exhibit enhanced energy metabolism and dysregulated immune signaling pathways, suggesting they may be better suited for metabolic-targeted therapies or immunotherapies. This model provides a practical tool for clinical risk stratification and personalized treatment selection, enabling clinicians to identify patients who may benefit from immune checkpoint inhibitors, metabolic inhibitors, or combination regimens. Furthermore, our study lays the foundation for translating this signature into a clinical assay to guide precision therapy for advanced PCa. These results underscore the necessity of developing precision treatment strategies, such as targeting metabolic vulnerabilities or tailoring regimens based on subtype-specific drug sensitivities.

With the advancement of single-cell technology, the exploration of the TME has become more common, helping to capture immune cell distribution within the tumor and to identify distinct cancer cell subgroups. Using our model, we screened high-risk cancer cell subgroups with high immune activity and employed the GSEA to explore their biological features. These results indicated that high-risk cancer cell subgroups could increase glycolysis rate and promote ATP production. Therefore, we hypothesized that the higher the proportion of high-risk cancer cell subgroups, the more vigorous their energy metabolism and the worse patient prognosis. Subsequent survival analysis based on the proportion of high-risk cell subgroups confirmed that patients with a higher proportion of these subgroups had a poorer prognosis. Although leveraging high-risk subpopulation proportions at single-cell resolution achieved statistically significant survival discrimination, the corresponding 1- and 5-year survival prediction AUC values (0.637 and 0.623, respectively) were substantially lower than those of the bulk RNA-seq model (0.854 and 0.889, respectively). This indicates considerable potential for enhancing the predictive capacity of single-cell-derived risk subpopulation metrics, likely constrained by limited sample sizes, cellular heterogeneity, or technical noise artifacts. Future investigations should integrate larger-scale single-cell datasets with multimodal data integration strategies to optimize model performance.

PCa is a hormone-dependent malignant tumor, and hormone receptor pathway-targeted therapies are the primary approaches to treatment. Second-generation androgen receptor inhibitors, such as apalutamide, have extensive clinical use. Although these drugs have improved the survival of patients with PCa, issues related to drug resistance remain a substantial challenge. Our findings suggest that different cancer cell subgroups exhibit varying levels of resistance to different anticancer drugs. Only subgroup 3 cancer cells were sensitive to apalutamide treatment, indicating the need to tailor treatment strategies based on the distribution of cancer cell subgroups. Future work will prioritize expanding the drug panel to include additional clinically critical therapeutics for mechanistic interrogation. Key resistance mechanisms identified herein will undergo rigorous validation in physiologically relevant preclinical models and/or clinical cohorts. Overall, we believe that interfering with cancer cell energy metabolism pathways using drugs that target specific metabolic enzymes might represent a potential therapeutic strategy. ATP-binding cassette (ABC) transporters, including ABCB1 and ABCG2, are recognized contributors to chemotherapy resistance^[51]^. However, our multi-omics analysis and genome-wide modeling did not identify these transporters as primary drivers of the immune-related risk subgroups. This indicates that within the immune-associated high-risk subtype identified in this study, resistance mechanisms are more likely influenced by dynamic changes in the immune microenvironment rather than conventional drug efflux systems. Our findings underscore the heterogeneity of drug resistance pathways in PCa and emphasize the importance of differentiating between immune-mediated and classical efflux-associated resistance mechanisms, which have significant implications for personalized treatment strategies.

Although resistance analyses have been conducted on different cancer cell subtypes, the efficacy of anticancer drugs in various clinical cancer cell subtypes remains a subject for further investigation. It should be noted that metabolic pathway identification in this study primarily relied on transcriptomic data, without orthogonal validation methods like metabolomics, which may affect result accuracy. In the future, we plan to further investigate the relationship between the immune microenvironment and resistance mechanisms, and integrate metabolomics and other experimental approaches in future work to further validate and refine metabolic pathway alterations in PCa subtypes. We will integrate additional transcriptomic data and scRNA-seq data to analyze drug-resistant characteristics across distinct immune subtypes. This research will enhance our understanding of the PCa immune microenvironment and its impact on therapeutic responses within a broader context, thereby providing novel insights for developing more effective treatment strategies. Furthermore, we will evaluate the sensitivity of various cellular clusters to specific anticancer agents by assessing their proliferative capacity. Validation experiments will investigate the differential expression of resistance-associated genes at both the mRNA and protein levels across these clusters. Cells will be exposed to gradient concentrations of anticancer drugs to compare IC50 values among subpopulations, thus analyzing the heterogeneity in drug resistance. Importantly, metabolic intervention may serve as a critical breakthrough based on our identified gene signatures. For instance, inhibiting the glycolytic pathway in tumor cells could attenuate their survival capacity. Notably, we observed a significant enrichment of muscle cell pathways, and we posit that this phenomenon may reflect distributional differences in stromal components (e.g., myofibroblasts) or processes such as epithelial-mesenchymal transition across risk subtypes within tumor tissues, although current evidence does not provide direct mechanistic support. Given the complexity of multi-omics data and the heterogeneity of the TME, the biological significance of this observation warrants further investigation through subsequent experiments and higher-resolution studies. We therefore present this finding as a novel observation that merits attention, and recommend future research to elucidate its underlying mechanisms and prognostic implications in PCa. We integrated drug resistance data from the GDSC database with PCa cell line expression profiles. These findings should be regarded as hypothesis-generating, offering preliminary insights that require validation through further experimentation and clinical investigation. While different cell subpopulations exhibited variable tolerability and sensitivity profiles to the four anticancer agents, suggesting potential differential optimal drug selection for specific subtypes, these results remain exploratory and warrant confirmation in future studies. Additionally, drug-resistant tumor cells may evade immune surveillance; thus, immune checkpoint inhibitors or other immunomodulatory therapies might partially restore antitumor immune activity. Another pivotal strategy involves combination therapies, as distinct resistant subtypes may resist monotherapies but could be effectively targeted through combined androgen receptor pathway inhibitors with metabolic interventions or immunotherapies. Although the current study has not yet experimentally validated these hypotheses, future research will systematically explore the feasibility of these therapeutic approaches.

In conclusion, this study delineates the intricate interplay between the dynamics of the immune microenvironment, genomic instability, and the heterogeneity of drug resistance in PCa. By integrating multi-omics data, we established a prognostic model that not only stratifies patients into high- and low-risk groups but also highlights subtype-specific drug resistance patterns. These results underscore the necessity for precision strategies that consider resistance mechanisms, such as targeting metabolic dependencies or tailoring therapies based on subtype-specific drug sensitivity profiles. Future studies should focus on translating these insights into combinatorial regimens to overcome resistance and enhance outcomes in advanced PCa.

## DECLARATIONS

### Availability of data and materials

All data relevant to the study are included in the article or uploaded as supplemental information. Data from the TCGA repository (https://www.cancer.gov/tcga) for the PRAD cohort are available. Gene expression data from the GEO repository (https://www.ncbi.nlm.nih.gov/geo/) include the following datasets: GSE46602, GSE70769, and GSE116918. Data will be made available from the corresponding authors upon reasonable request. For any other inquiries regarding the data from this study, please contact the author Li C.

### Copyright

© The Author(s) 2025.
