# Supplementary figures and images for: Integrated multi-omics profiling of immune microenvironment and drug resistance signatures for precision prognosis in prostate cancer
Source: Cancer Drug Resist. 2025 Jun 25;8:31. doi: 10.20517/cdr.2025.47 (PMC12366425; doi:10.20517/cdr.2025.47)

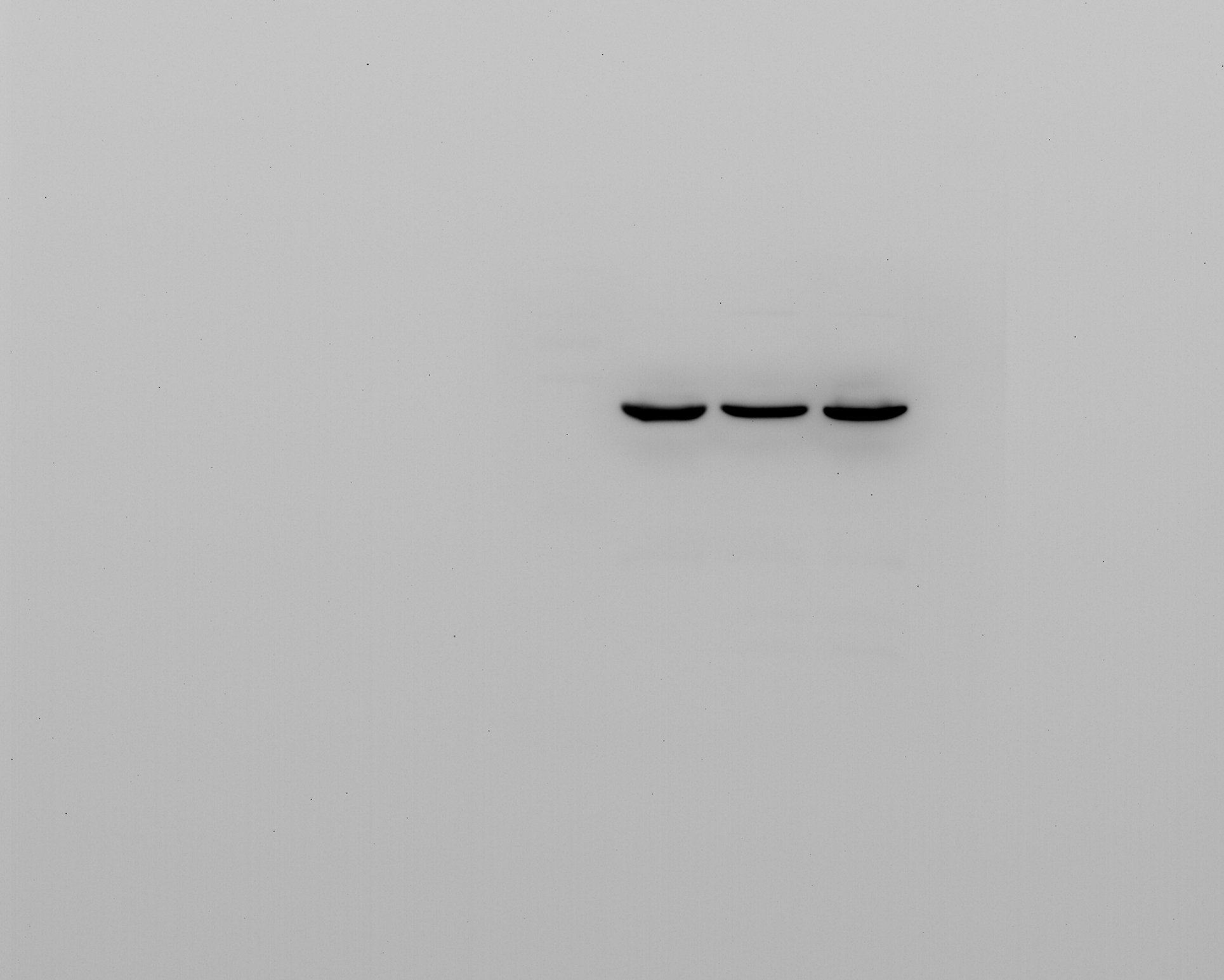

Supplement: Supplementary file 1 [file cdr-8-31-SupplementaryMaterials.zip › Western Blot/Actin/2023-07-26 10ú║16ú║15 _18.tif]

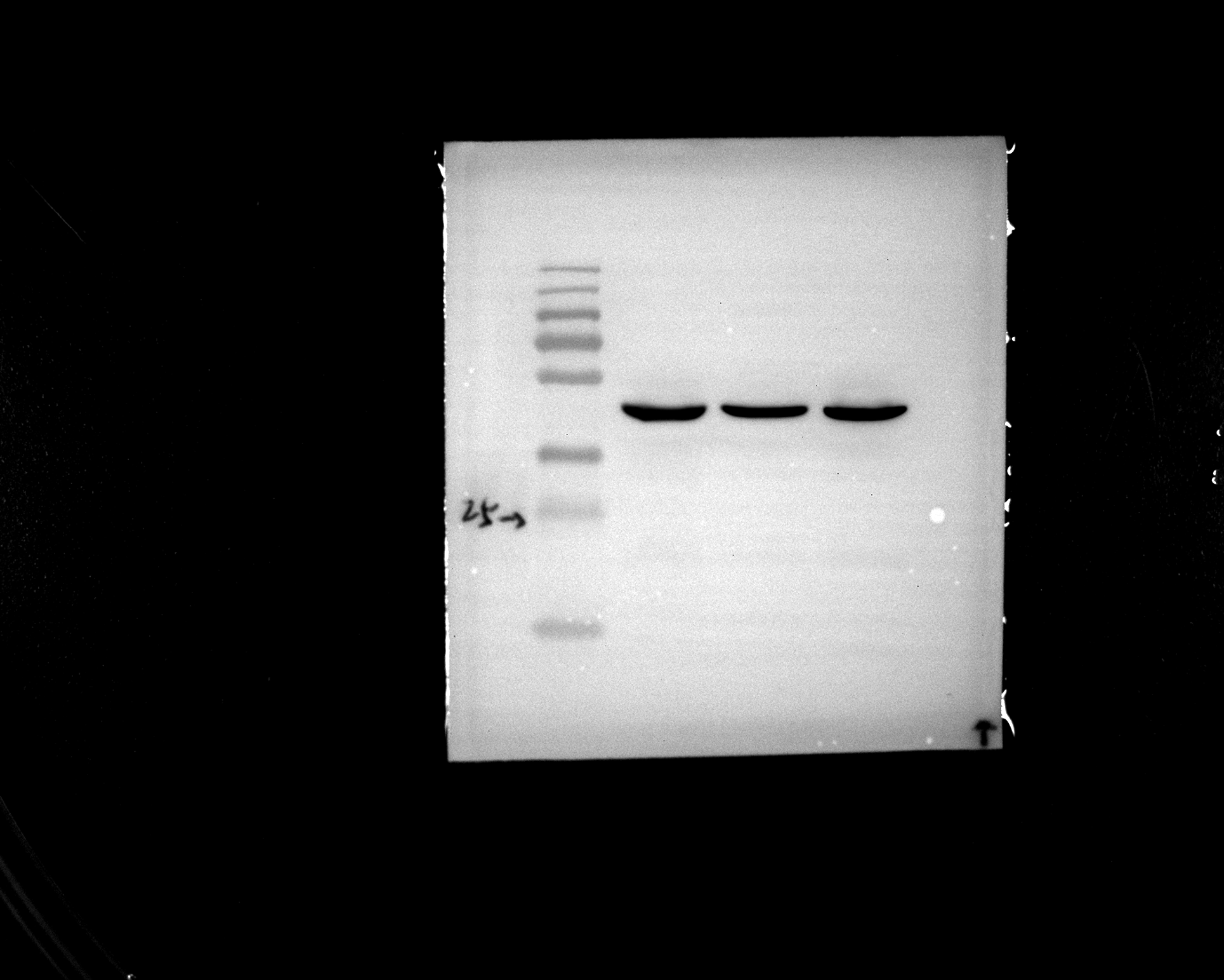

Supplement: Supplementary file 1 [file cdr-8-31-SupplementaryMaterials.zip › Western Blot/Actin/2023-07-26 10ú║16ú║15 _18merger.tif]

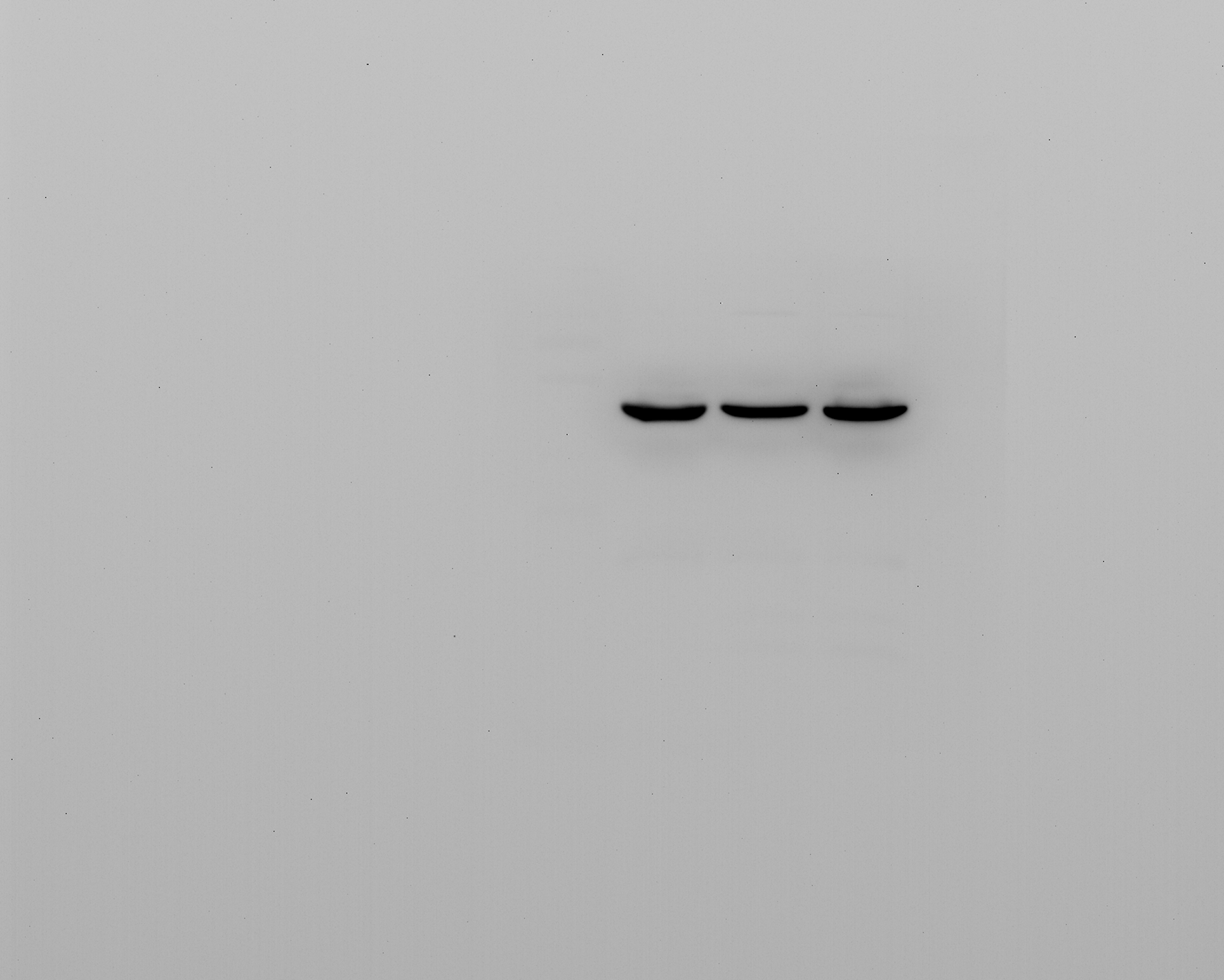

Supplement: Supplementary file 1 [file cdr-8-31-SupplementaryMaterials.zip › Western Blot/Actin/2023-07-26 10ú║16ú║18 _19 .tif]

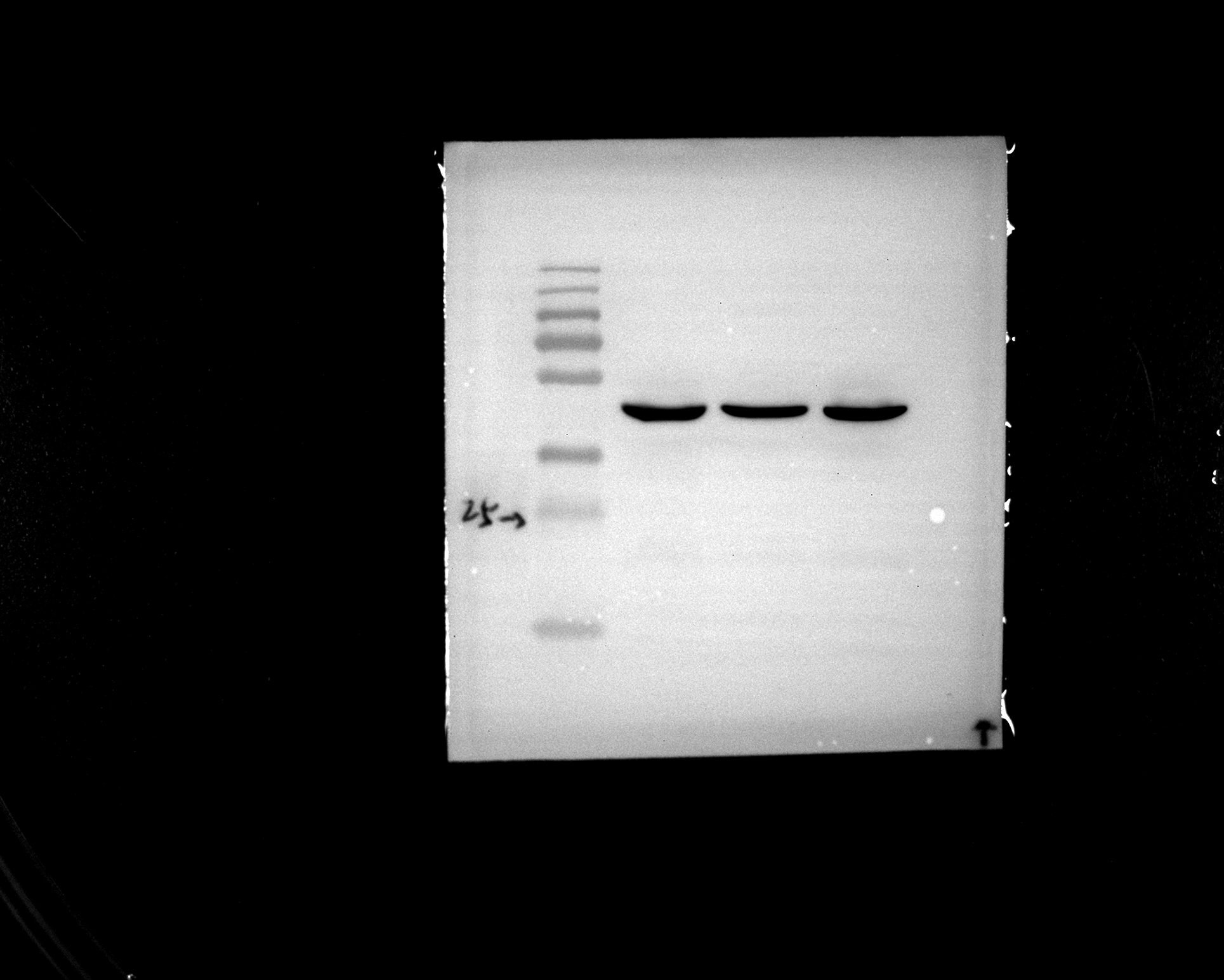

Supplement: Supplementary file 1 [file cdr-8-31-SupplementaryMaterials.zip › Western Blot/Actin/2023-07-26 10ú║16ú║18 _19merger.tif]

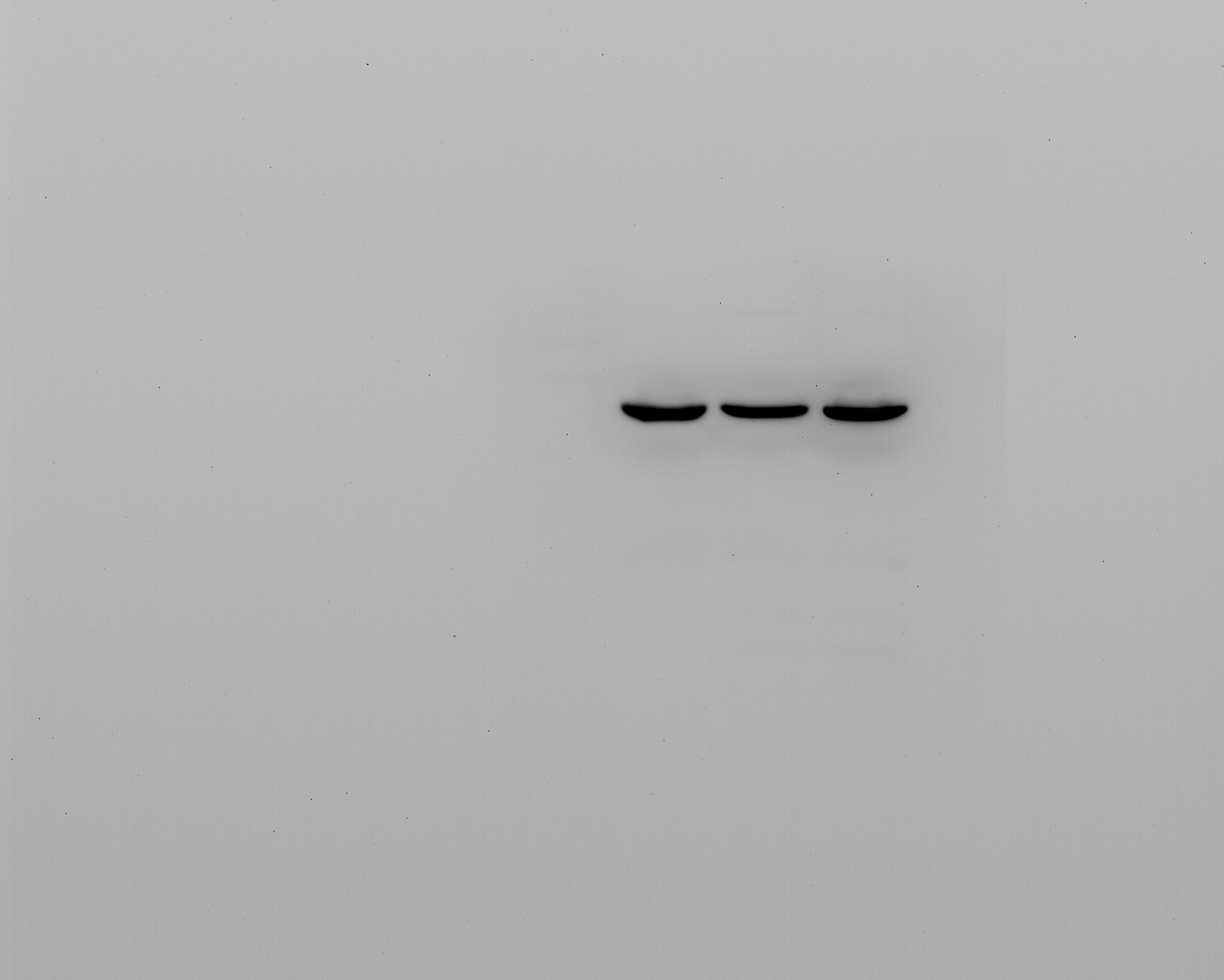

Supplement: Supplementary file 1 [file cdr-8-31-SupplementaryMaterials.zip › Western Blot/Actin/2023-07-26 10ú║16ú║20 _20.tif]

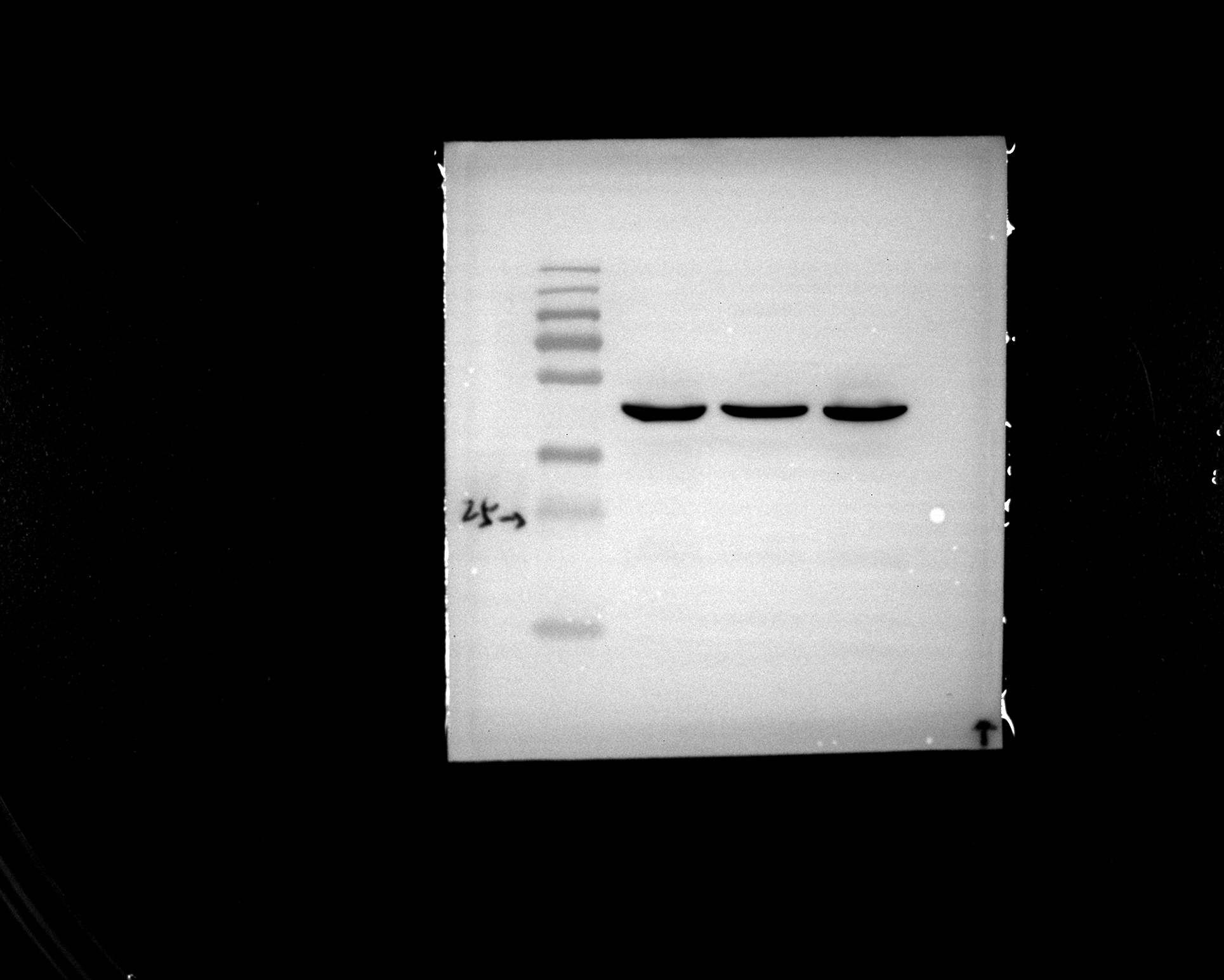

Supplement: Supplementary file 1 [file cdr-8-31-SupplementaryMaterials.zip › Western Blot/Actin/2023-07-26 10ú║16ú║20 _20merger.tif]

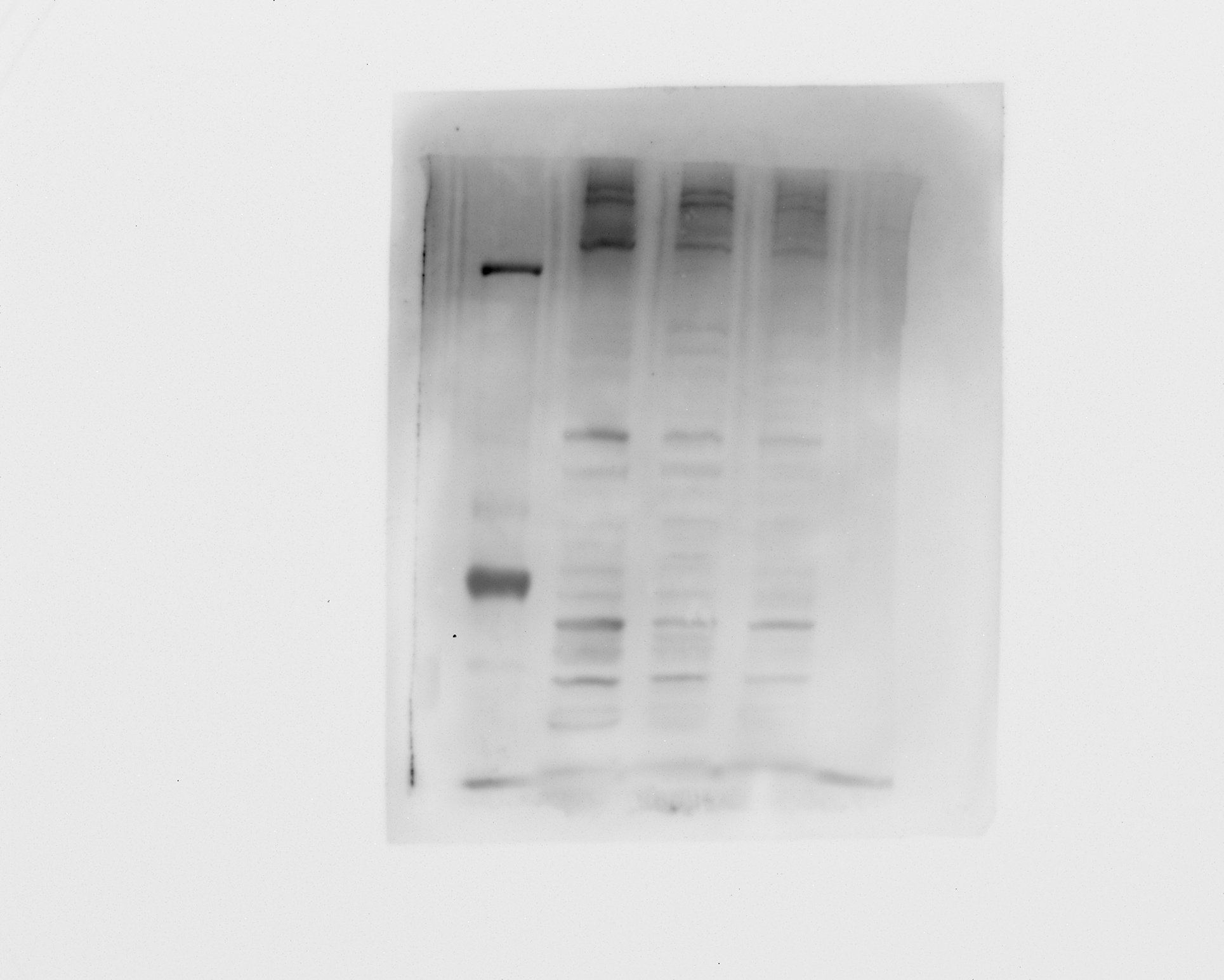

Supplement: Supplementary file 1 [file cdr-8-31-SupplementaryMaterials.zip › Western Blot/MUC5B/2023-07-25 11ú║36ú║41 _5.tif]

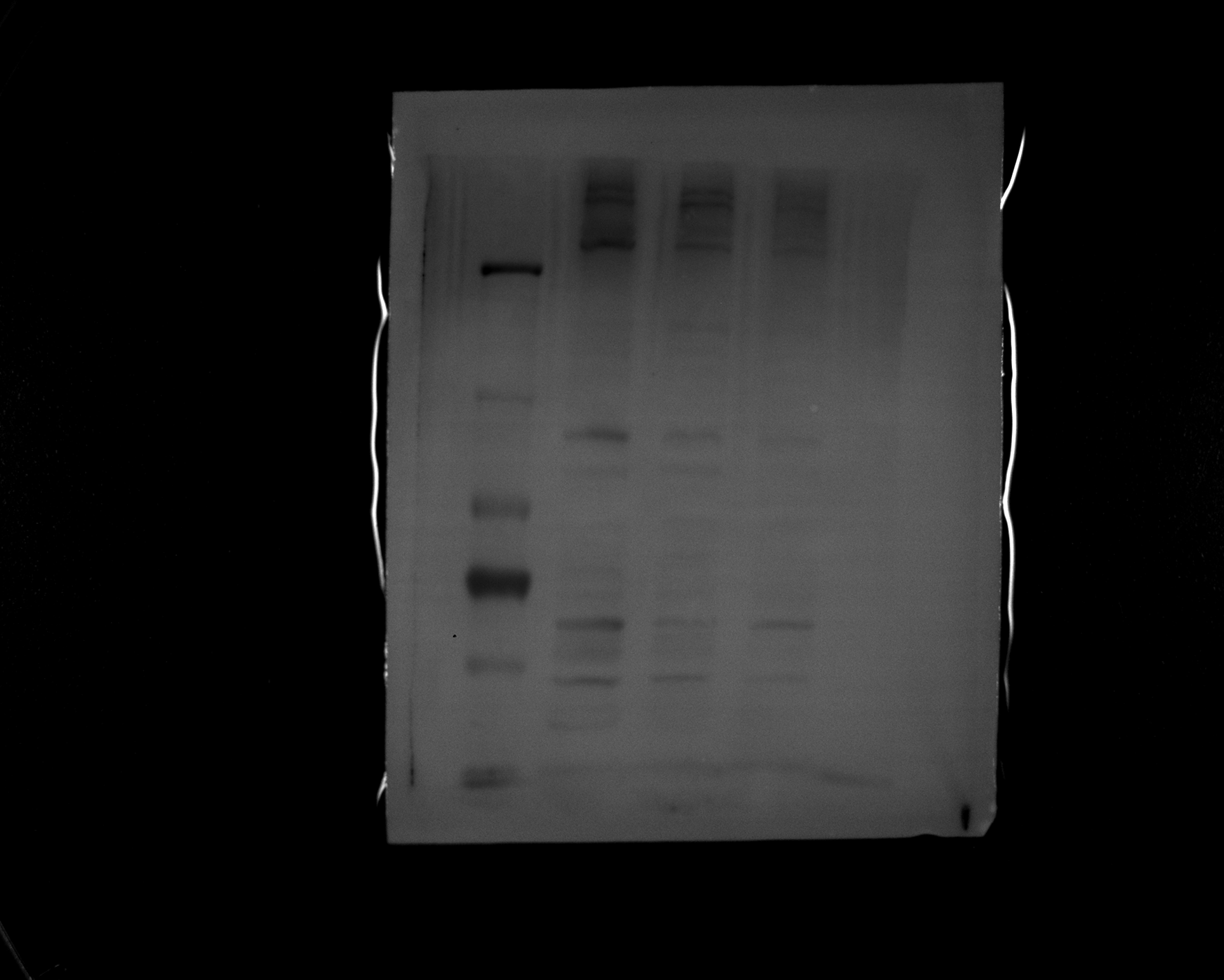

Supplement: Supplementary file 1 [file cdr-8-31-SupplementaryMaterials.zip › Western Blot/MUC5B/2023-07-25 11ú║36ú║41 _5merger.tif]

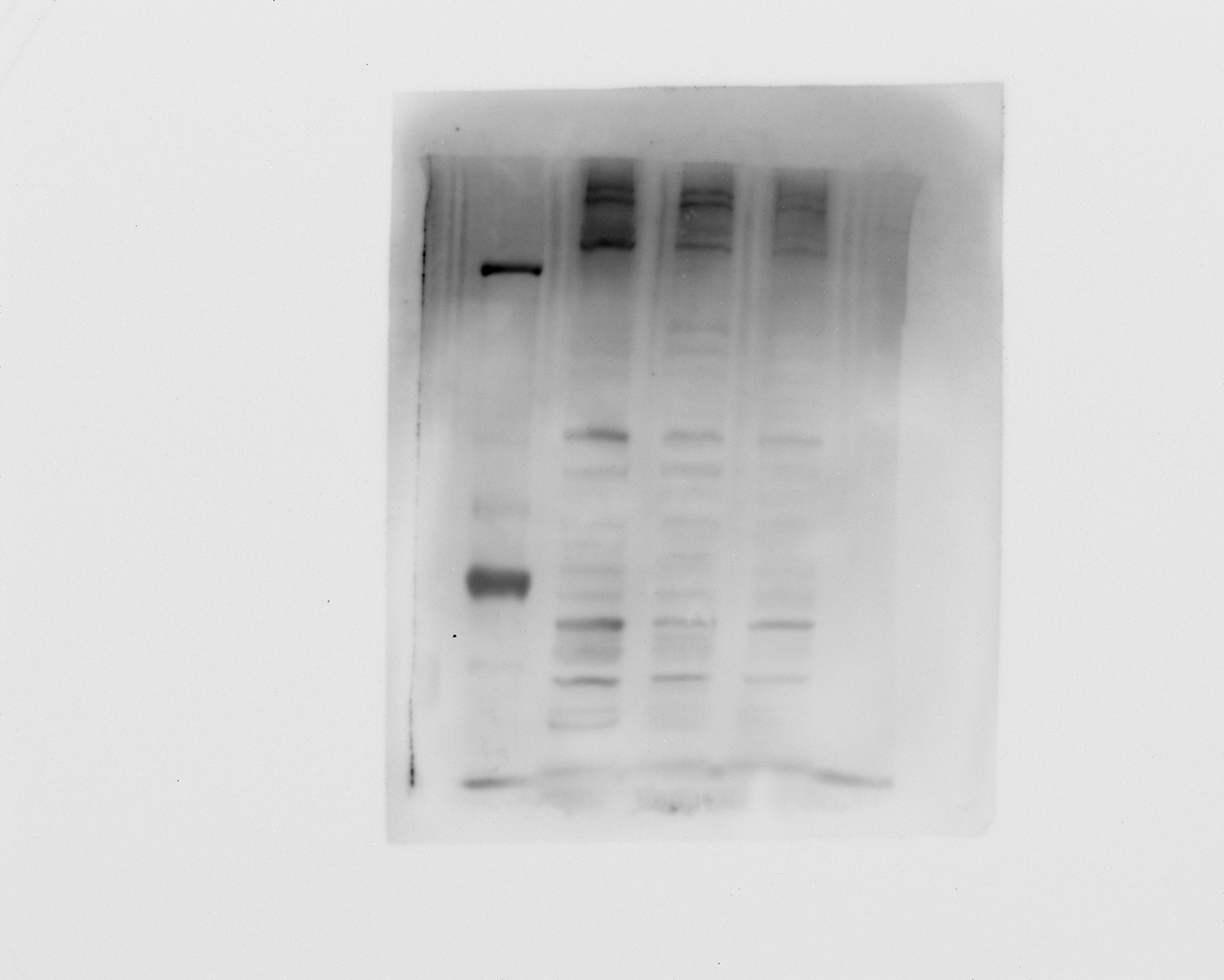

Supplement: Supplementary file 1 [file cdr-8-31-SupplementaryMaterials.zip › Western Blot/MUC5B/2023-07-25 11ú║38ú║04 _6.tif]

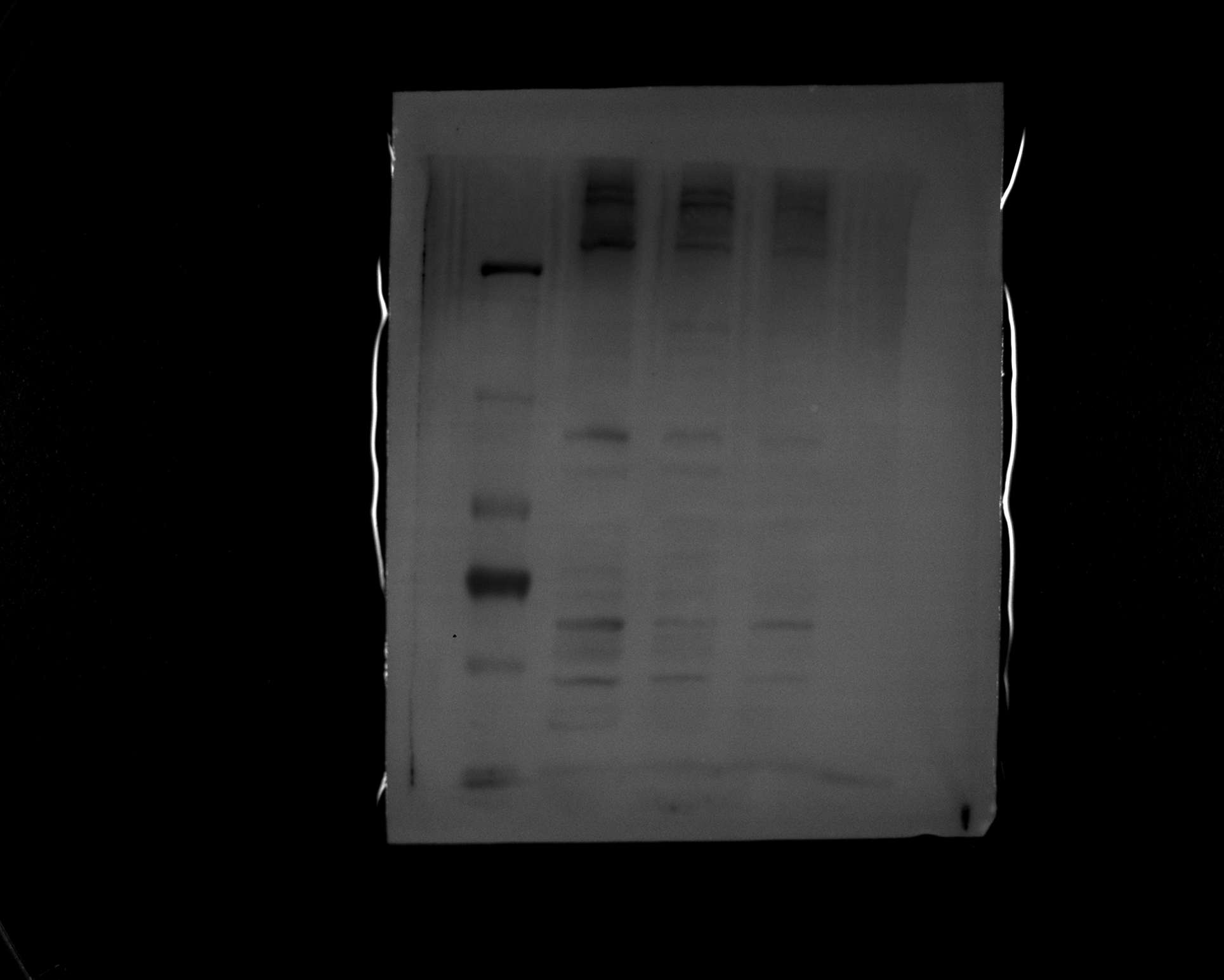

Supplement: Supplementary file 1 [file cdr-8-31-SupplementaryMaterials.zip › Western Blot/MUC5B/2023-07-25 11ú║38ú║04 _6merger.tif]

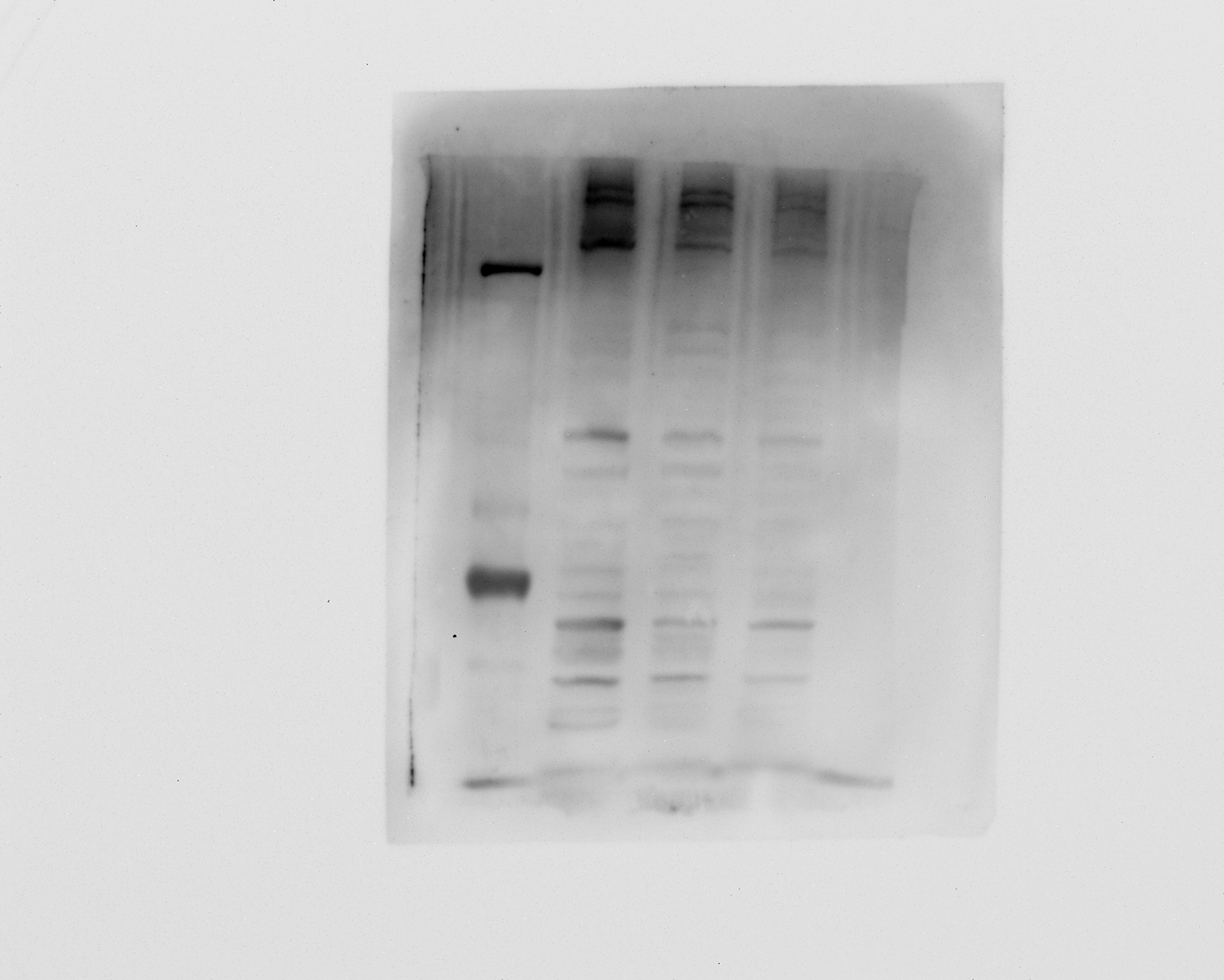

Supplement: Supplementary file 1 [file cdr-8-31-SupplementaryMaterials.zip › Western Blot/MUC5B/2023-07-25 11ú║39ú║27 _7 .tif]

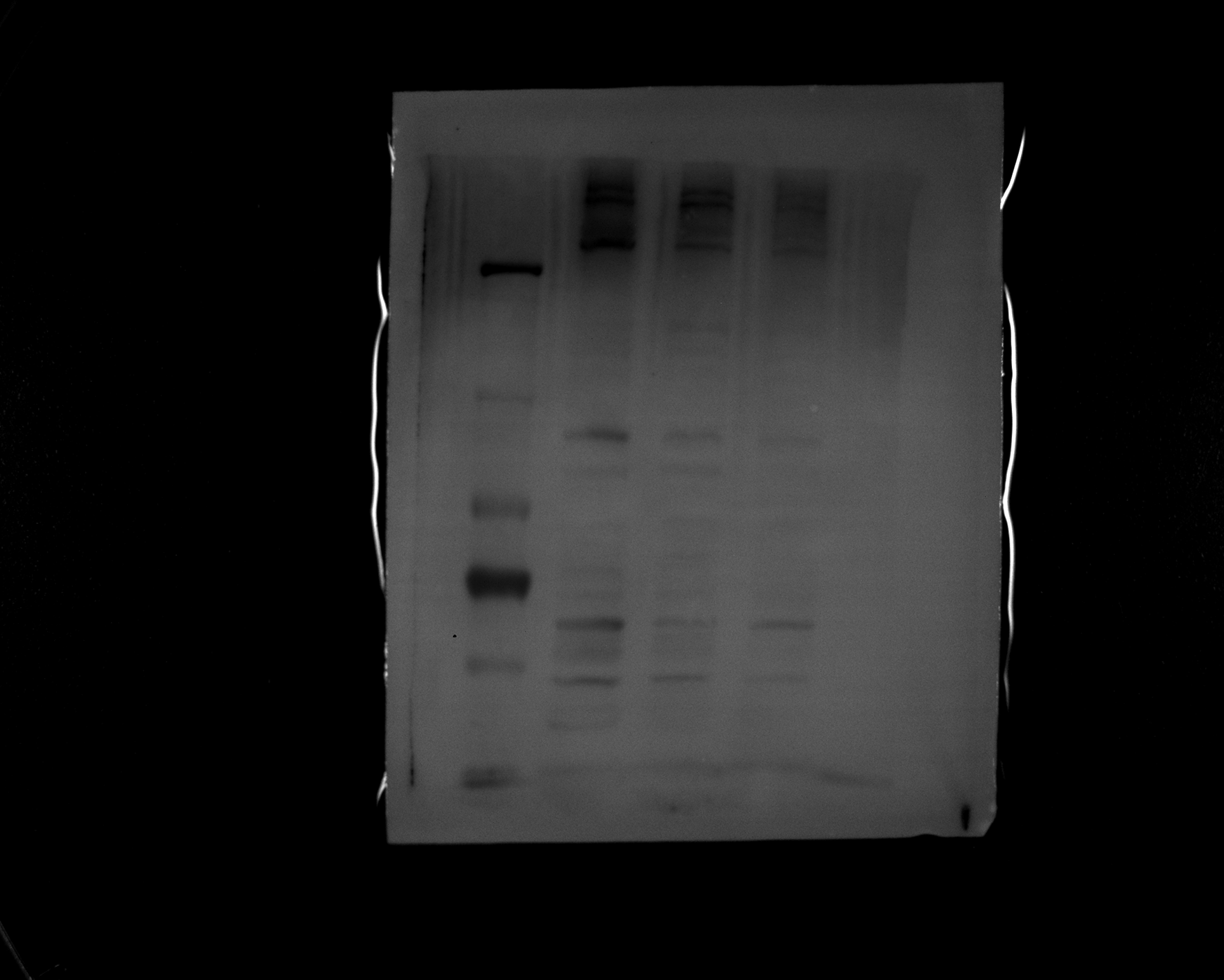

Supplement: Supplementary file 1 [file cdr-8-31-SupplementaryMaterials.zip › Western Blot/MUC5B/2023-07-25 11ú║39ú║27 _7merger.tif]

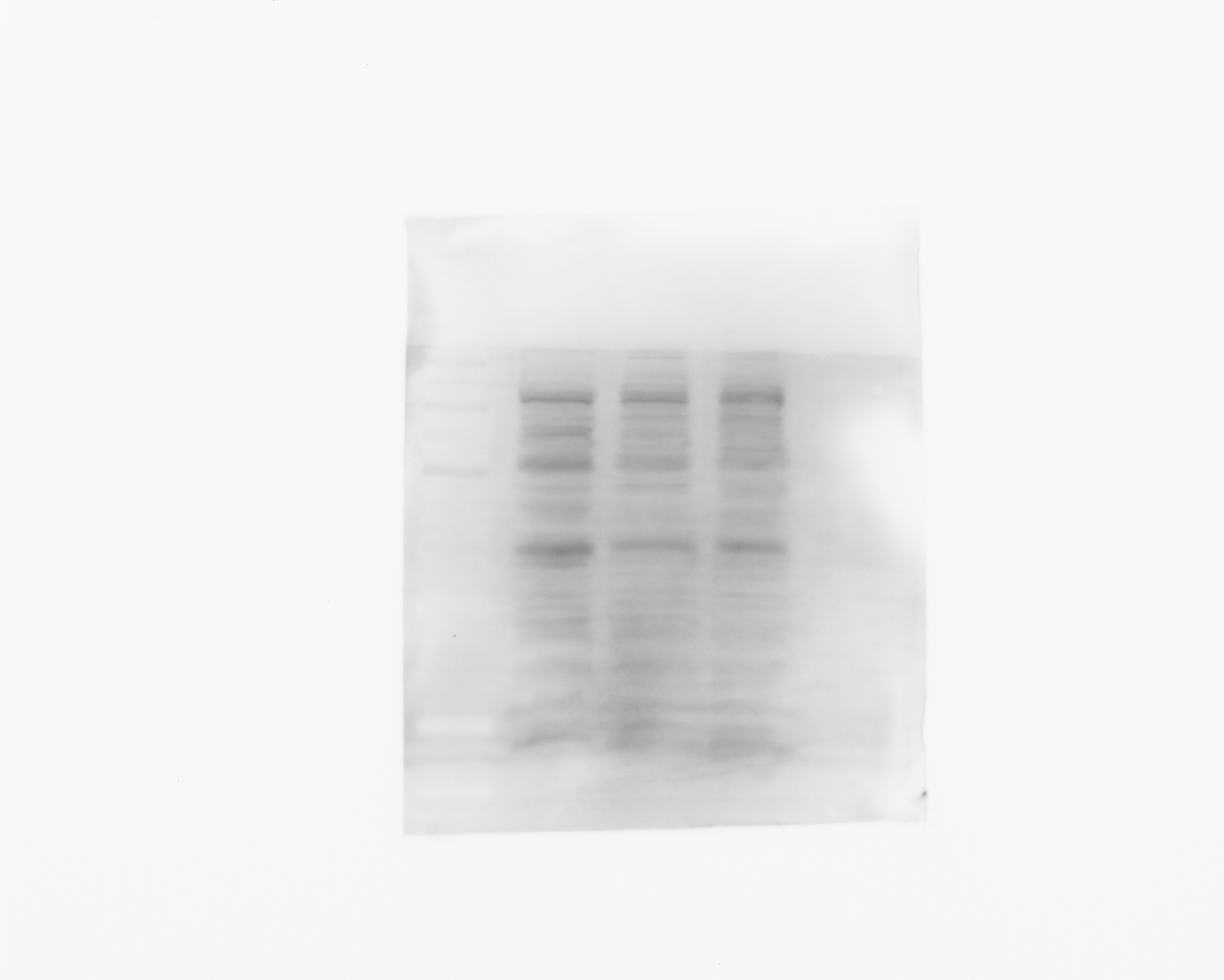

Supplement: Supplementary file 1 [file cdr-8-31-SupplementaryMaterials.zip › Western Blot/RAB33A/2023-07-25 14ú║29ú║53 _2.tif]

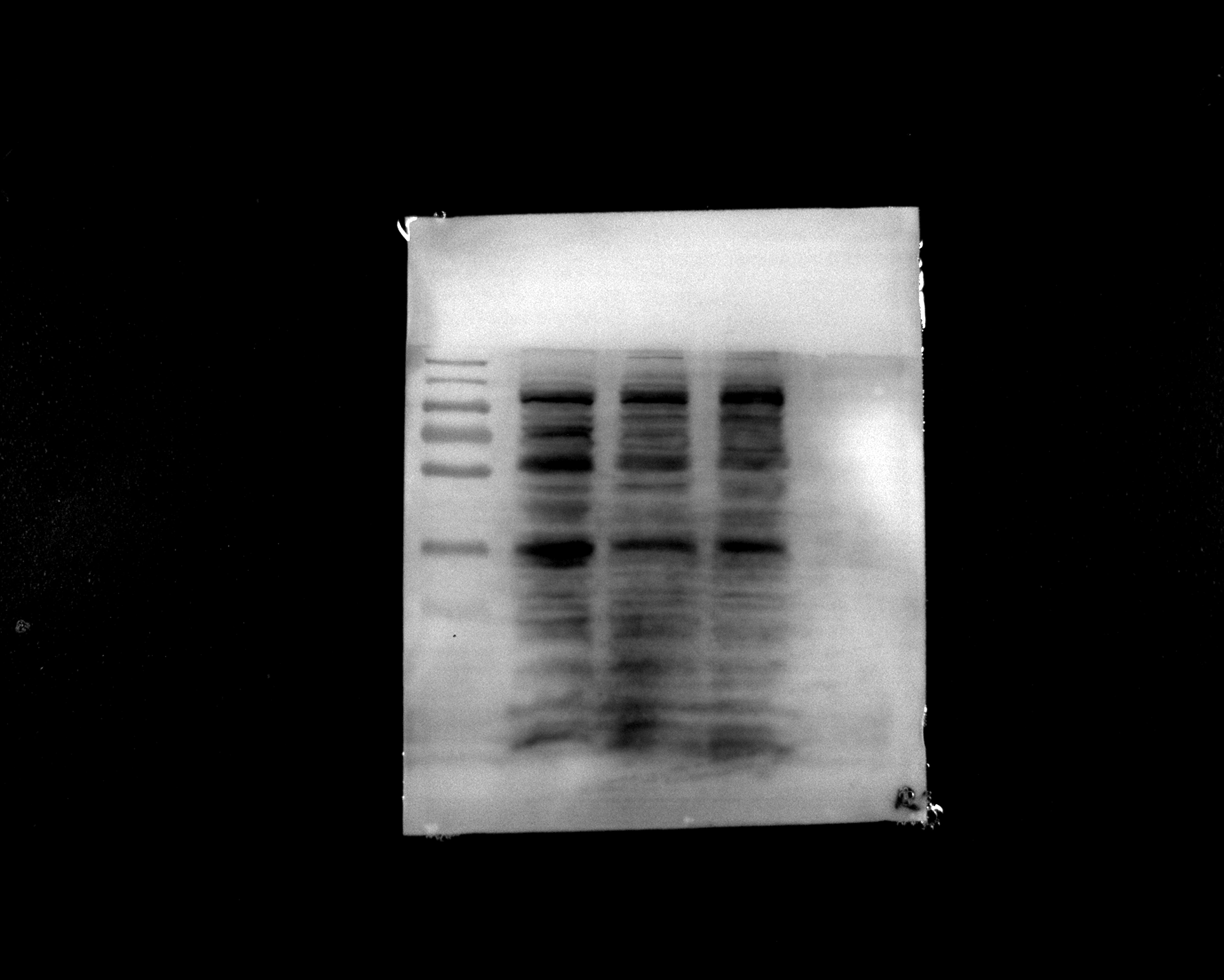

Supplement: Supplementary file 1 [file cdr-8-31-SupplementaryMaterials.zip › Western Blot/RAB33A/2023-07-25 14ú║29ú║53 _2merger.tif]

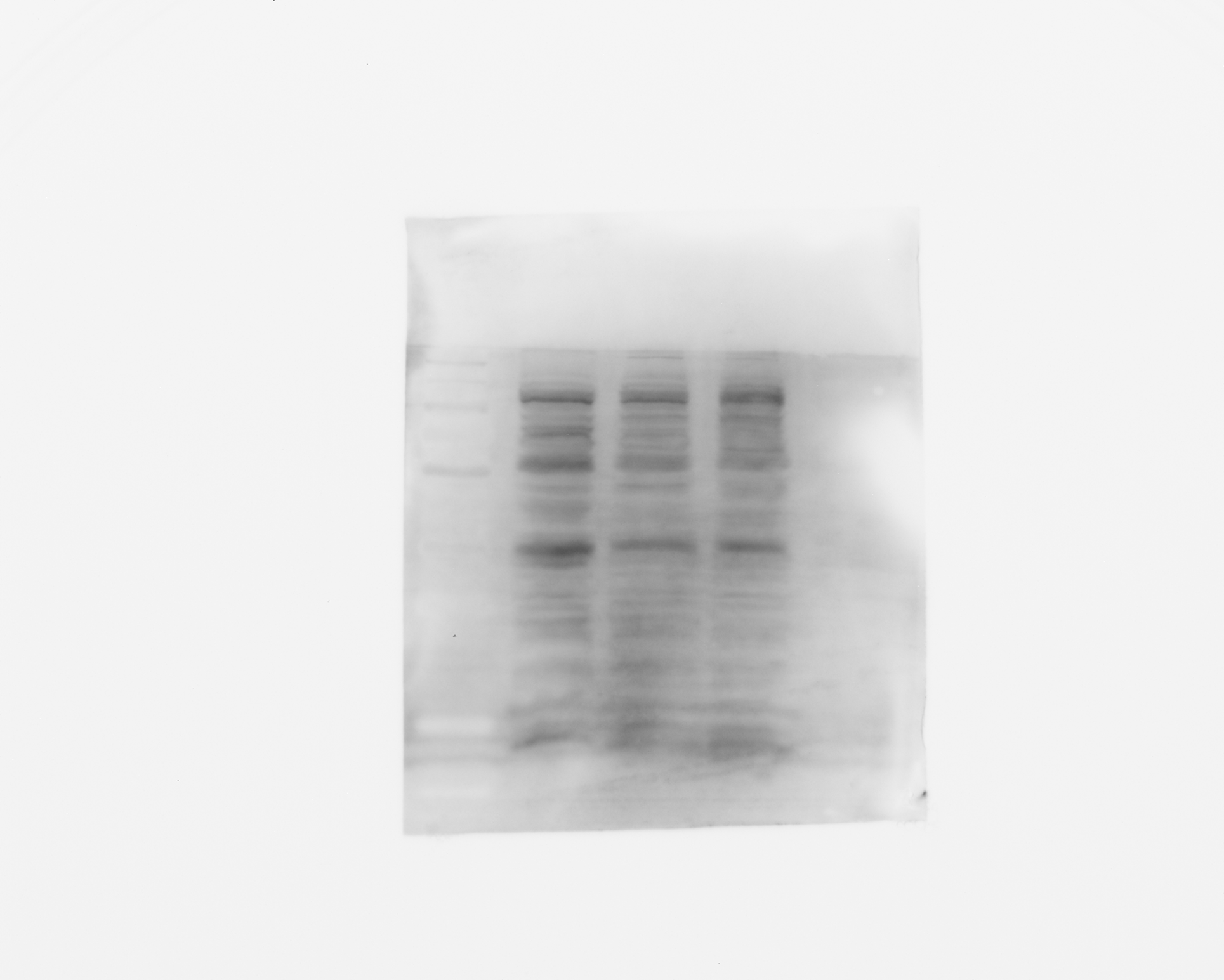

Supplement: Supplementary file 1 [file cdr-8-31-SupplementaryMaterials.zip › Western Blot/RAB33A/2023-07-25 14ú║30ú║14 _3.tif]

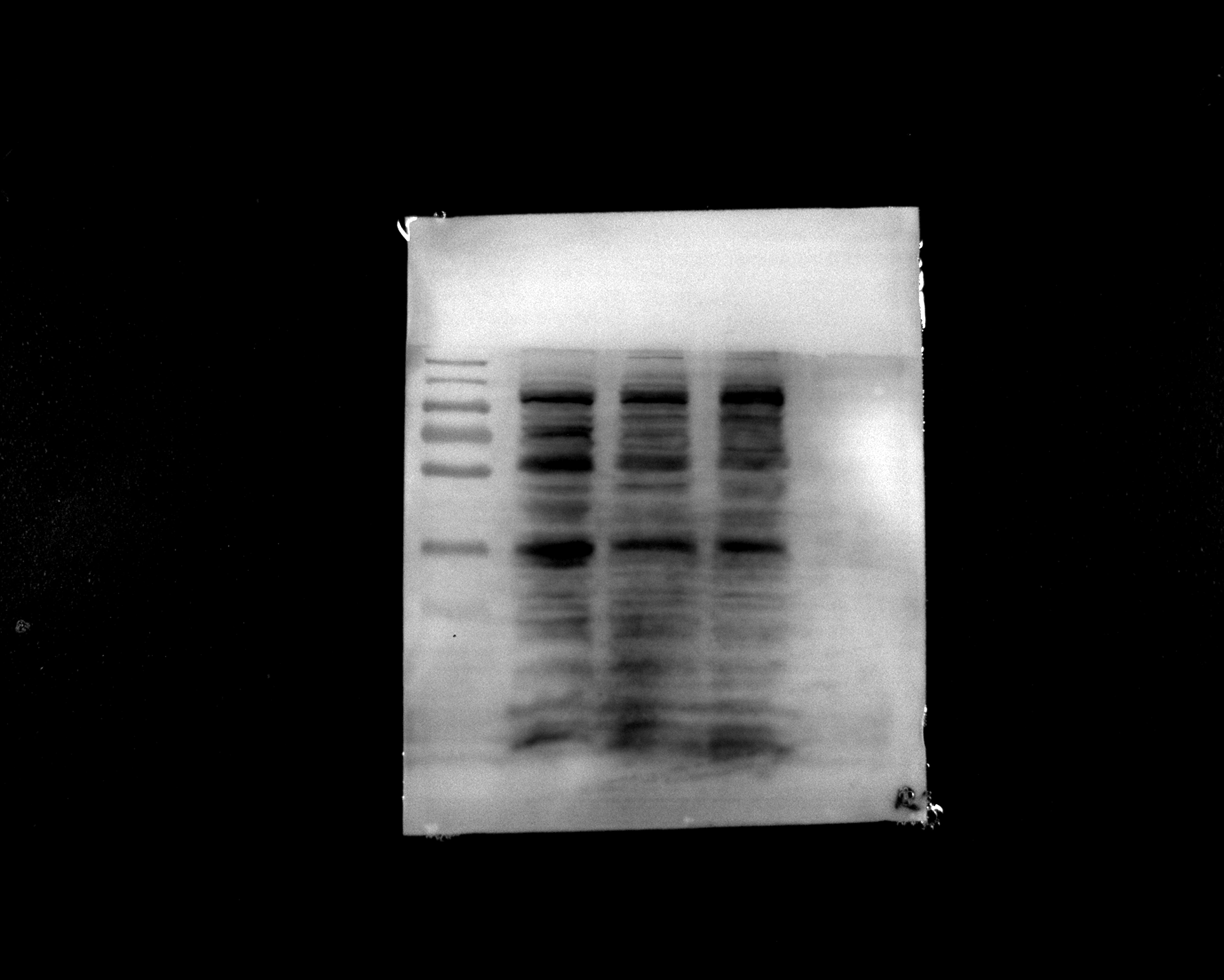

Supplement: Supplementary file 1 [file cdr-8-31-SupplementaryMaterials.zip › Western Blot/RAB33A/2023-07-25 14ú║30ú║14 _3merger.tif]

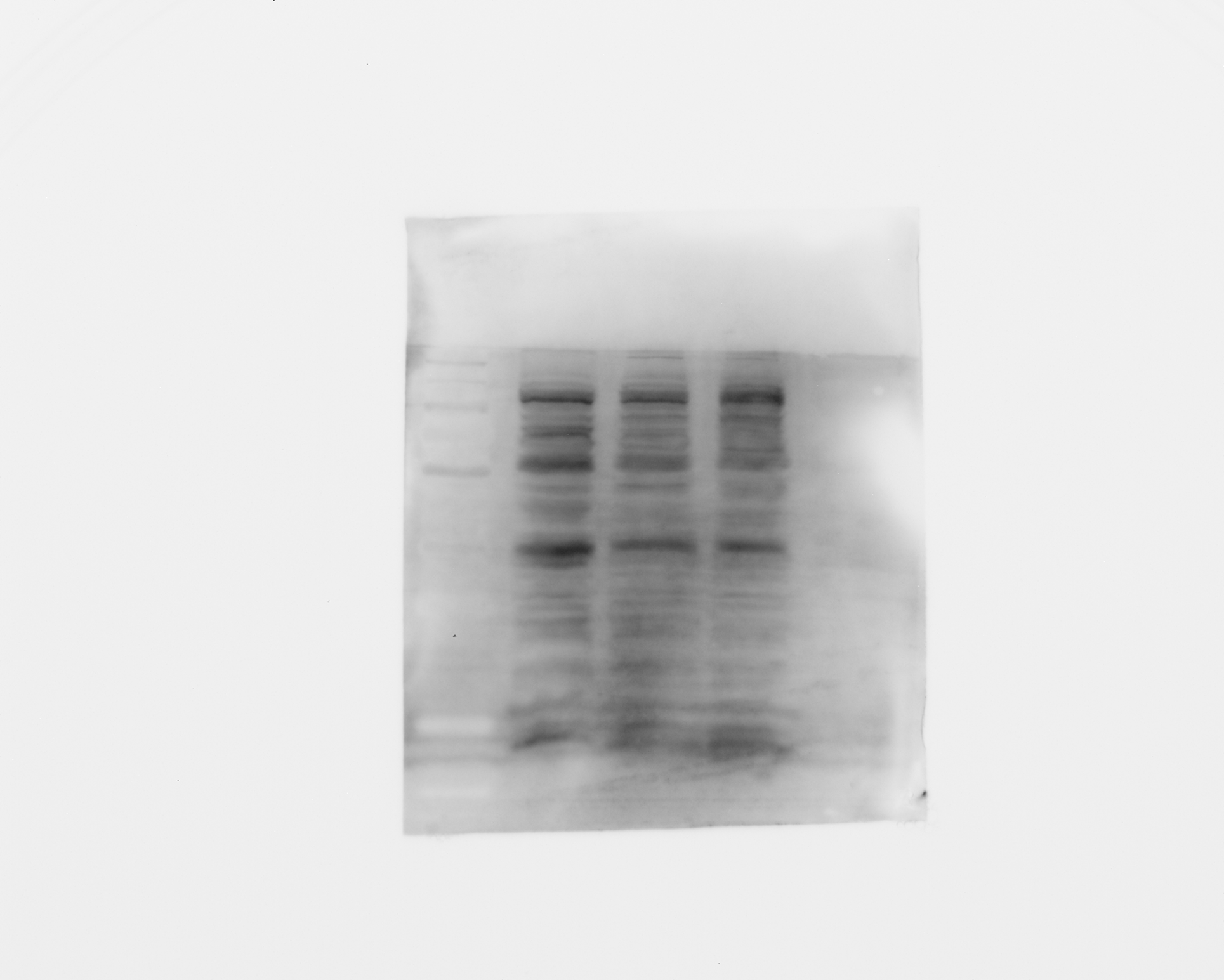

Supplement: Supplementary file 1 [file cdr-8-31-SupplementaryMaterials.zip › Western Blot/RAB33A/2023-07-25 14ú║30ú║35 _4 .tif]

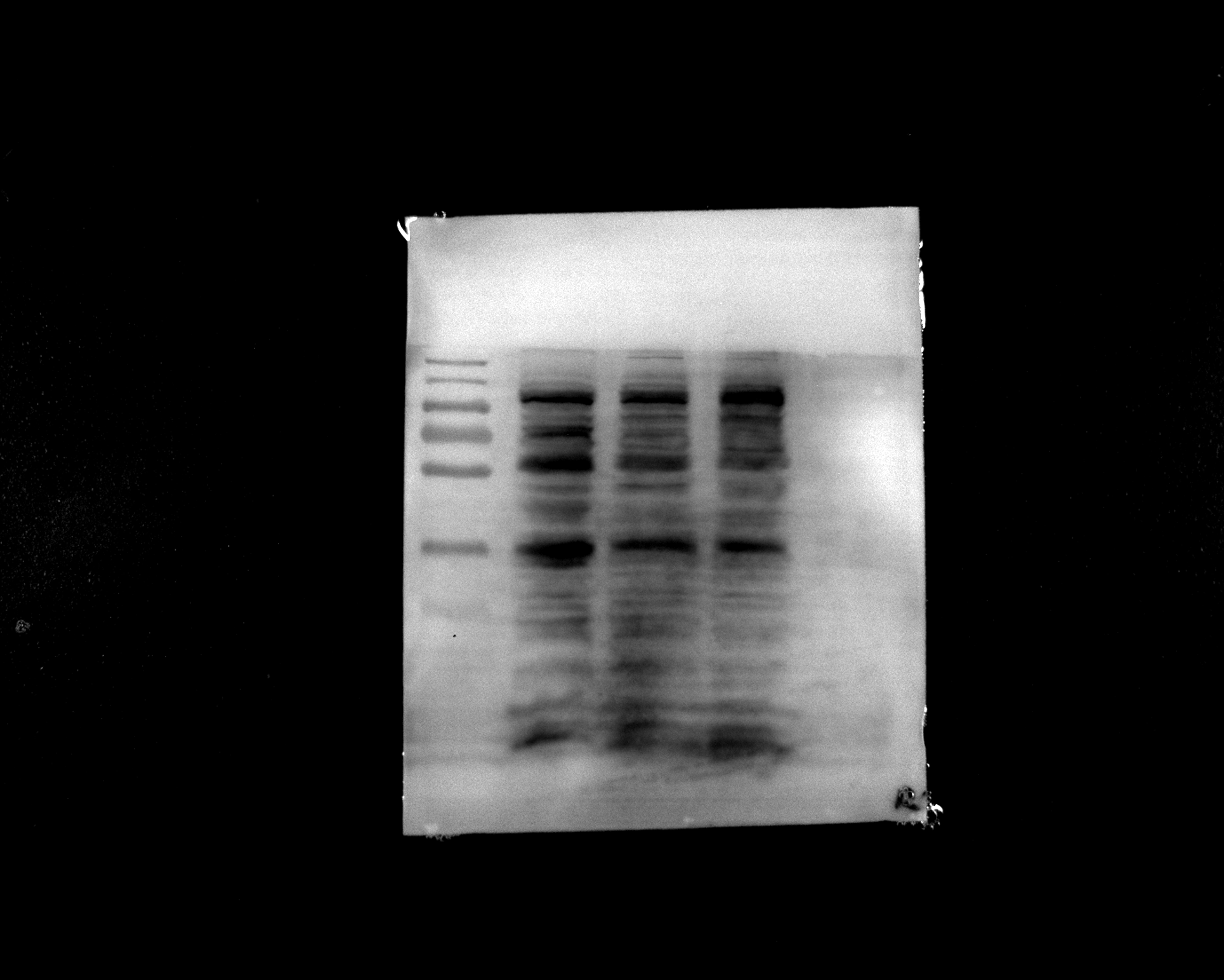

Supplement: Supplementary file 1 [file cdr-8-31-SupplementaryMaterials.zip › Western Blot/RAB33A/2023-07-25 14ú║30ú║35 _4merger.tif]

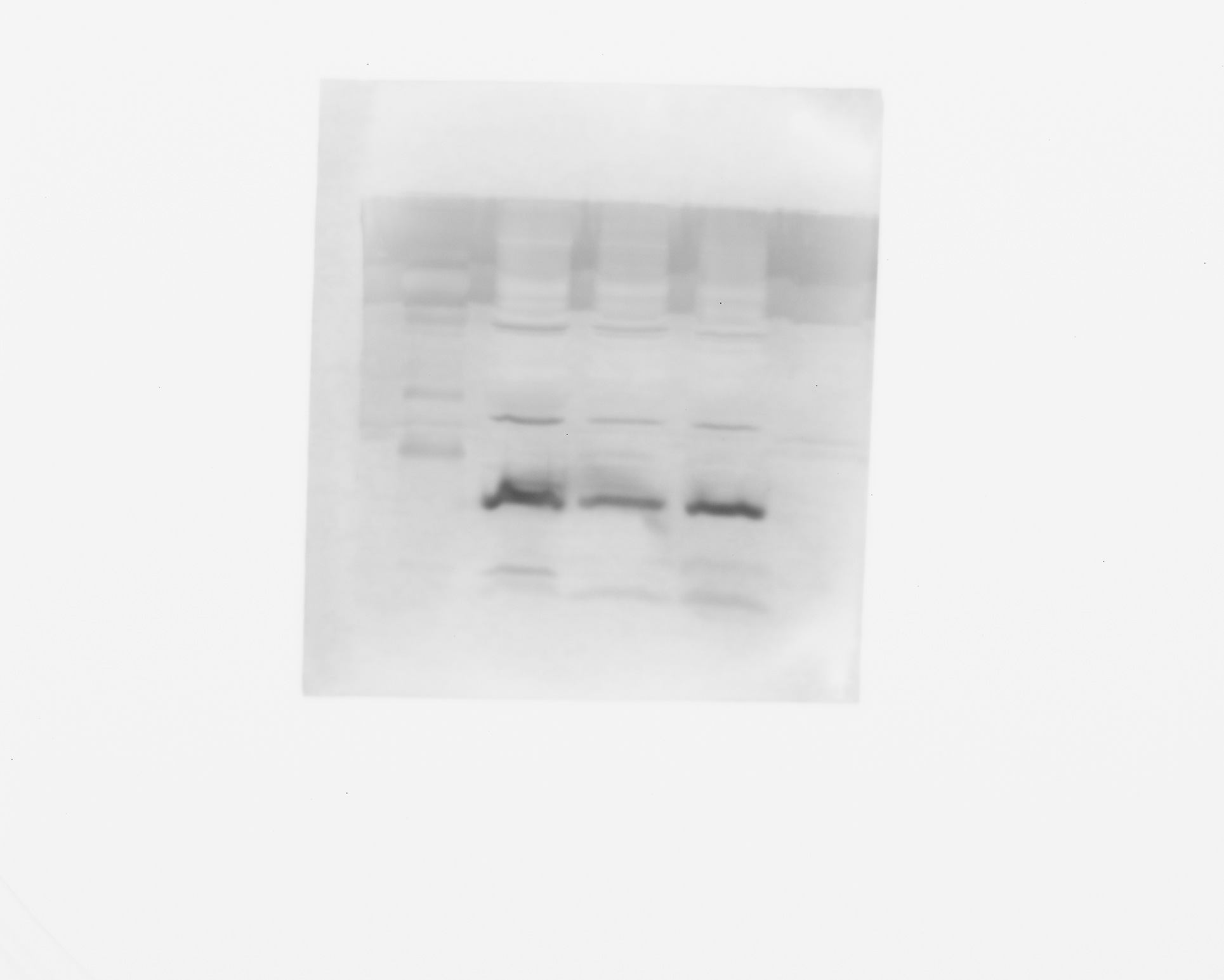

Supplement: Supplementary file 1 [file cdr-8-31-SupplementaryMaterials.zip › Western Blot/TREM1/2023-07-25 14ú║03ú║28 _3.tif]

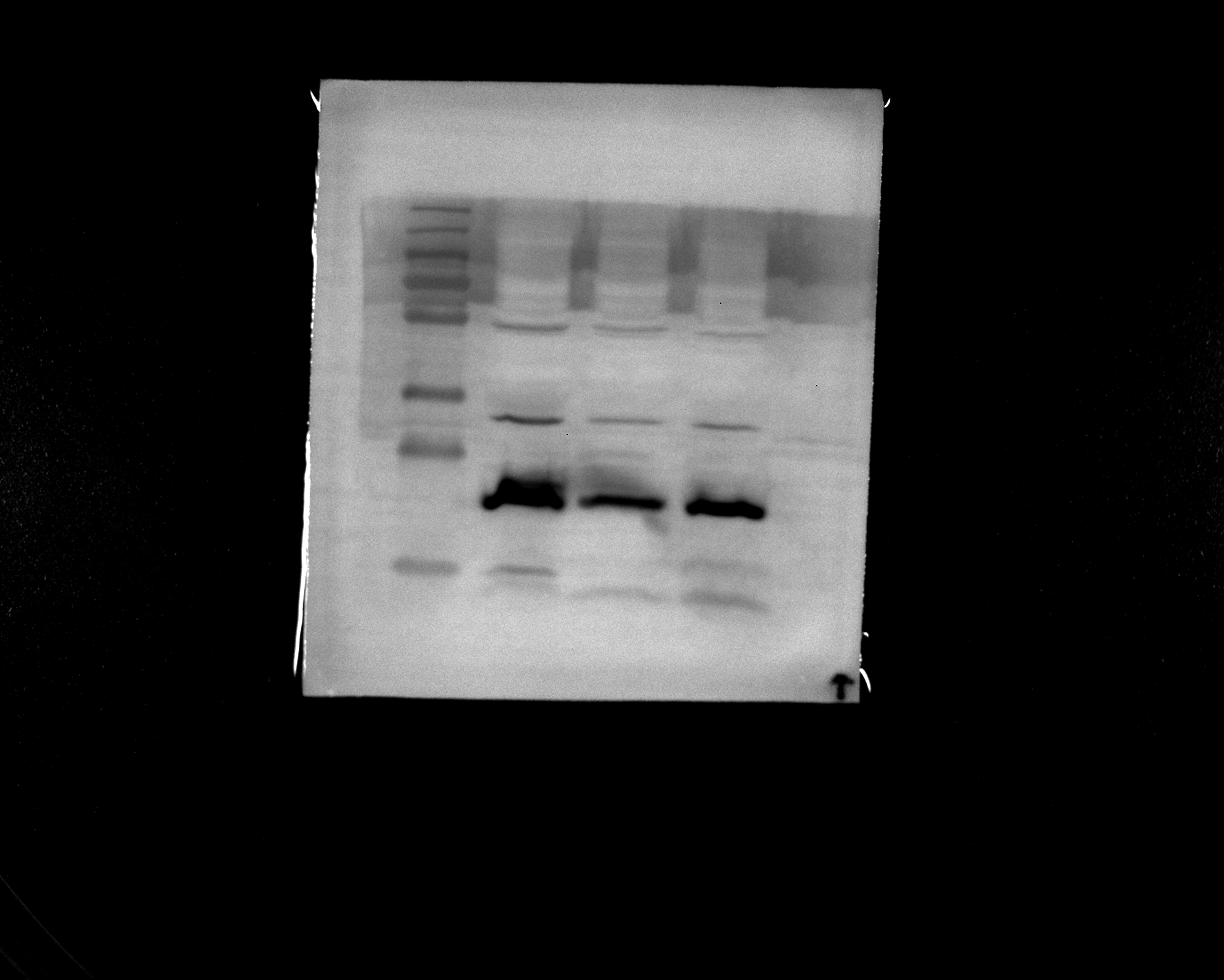

Supplement: Supplementary file 1 [file cdr-8-31-SupplementaryMaterials.zip › Western Blot/TREM1/2023-07-25 14ú║03ú║28 _3merger.tif]

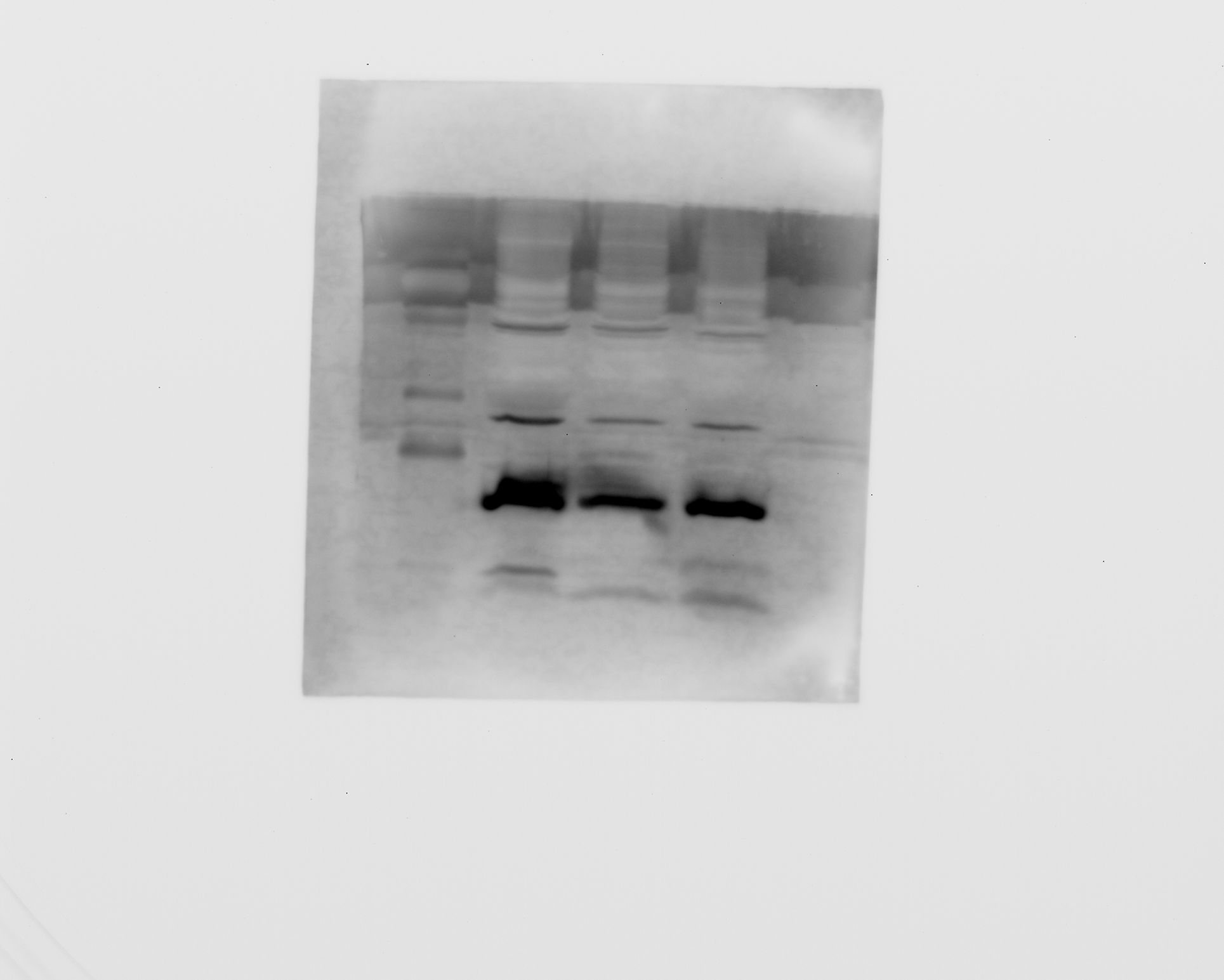

Supplement: Supplementary file 1 [file cdr-8-31-SupplementaryMaterials.zip › Western Blot/TREM1/2023-07-25 14ú║03ú║37 _7.tif]

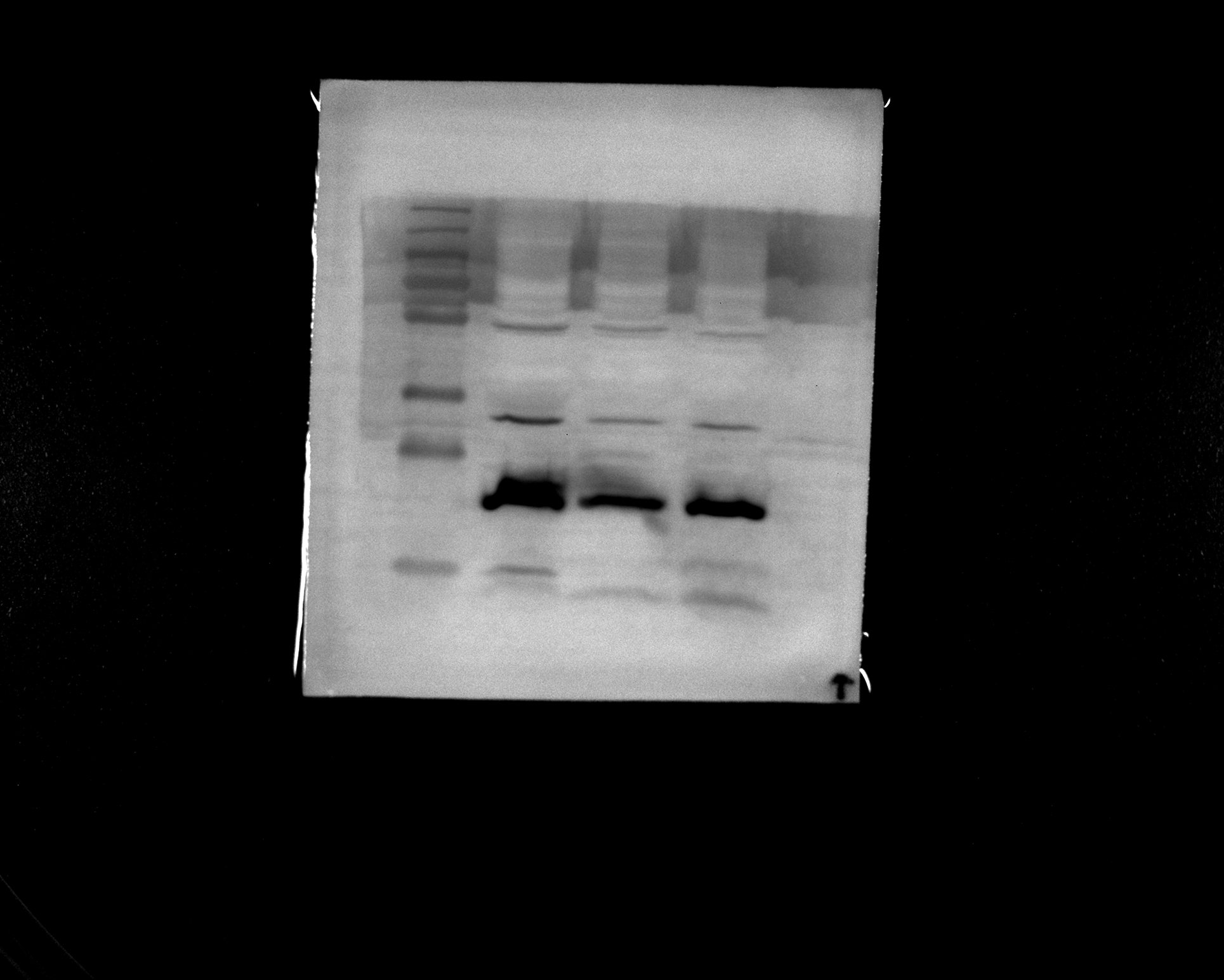

Supplement: Supplementary file 1 [file cdr-8-31-SupplementaryMaterials.zip › Western Blot/TREM1/2023-07-25 14ú║03ú║37 _7merger.tif]

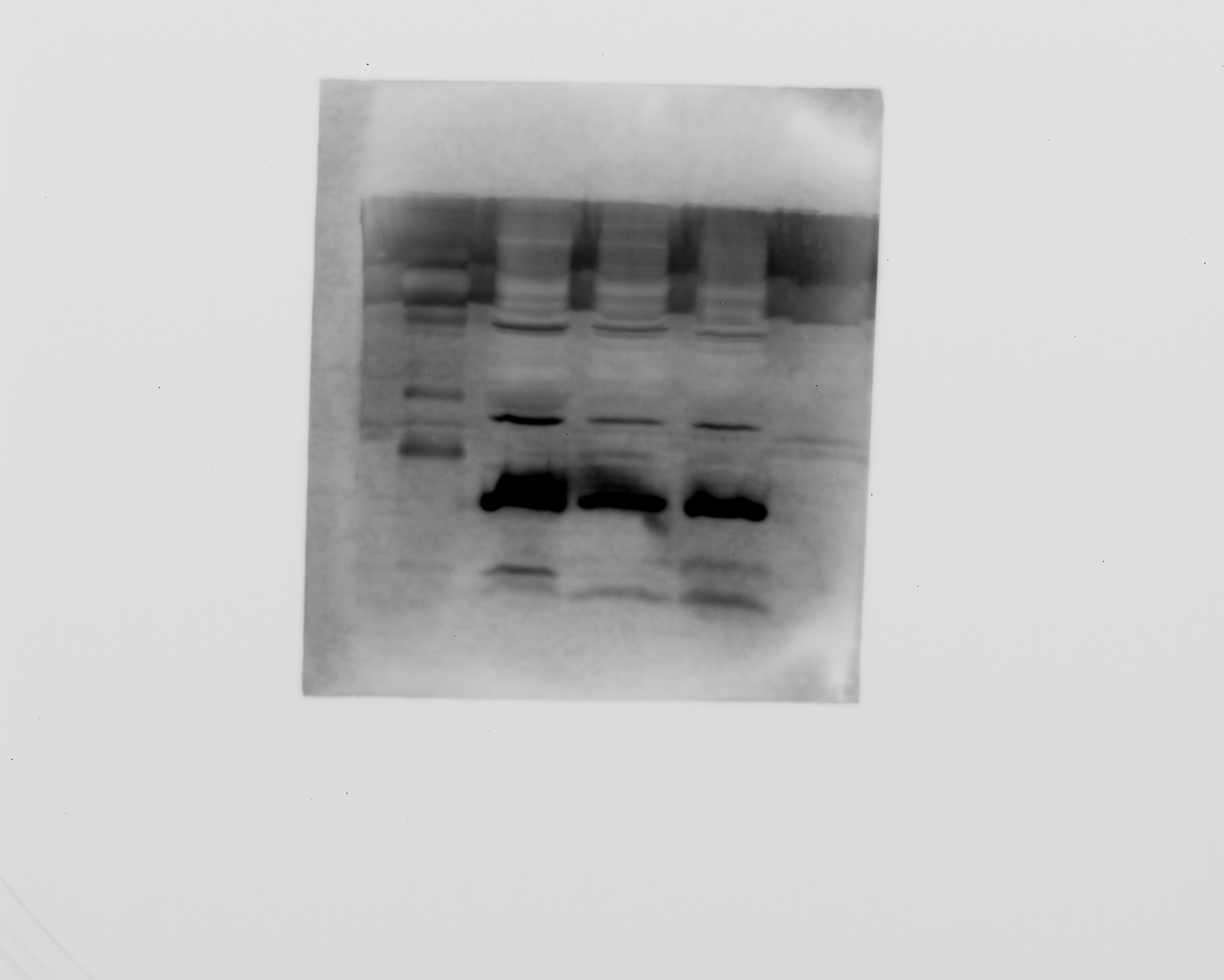

Supplement: Supplementary file 1 [file cdr-8-31-SupplementaryMaterials.zip › Western Blot/TREM1/2023-07-25 14ú║03ú║41 _9.tif]

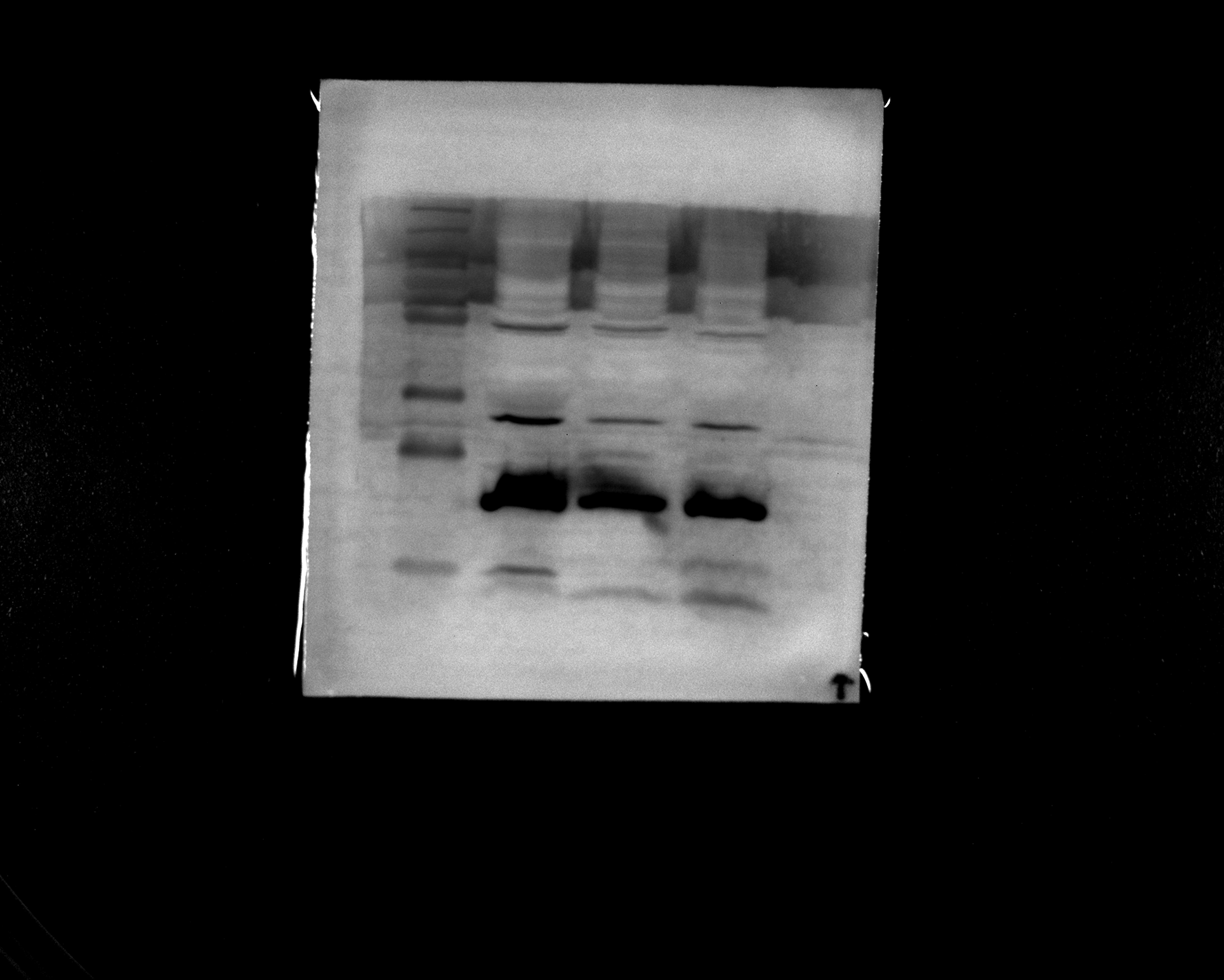

Supplement: Supplementary file 1 [file cdr-8-31-SupplementaryMaterials.zip › Western Blot/TREM1/2023-07-25 14ú║03ú║41 _9merger.tif]

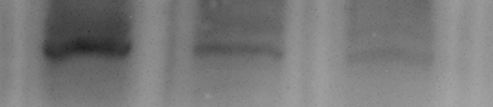

Supplement: Supplementary file 1 [file cdr-8-31-SupplementaryMaterials.zip › Western Blot/TREM1/Selected/2023-07-25 11ú║36ú║41 _5.tif]

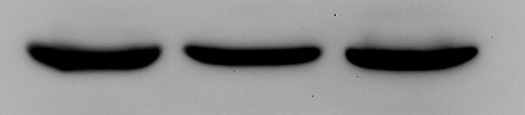

Supplement: Supplementary file 1 [file cdr-8-31-SupplementaryMaterials.zip › Western Blot/TREM1/Selected/Actin 2023-07-26 10ú║16ú║18 _19.tif]

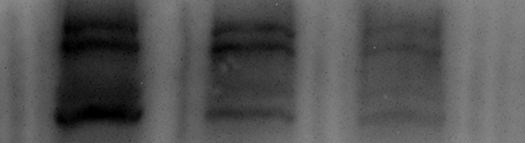

Supplement: Supplementary file 1 [file cdr-8-31-SupplementaryMaterials.zip › Western Blot/TREM1/Selected/MUC5 2023-07-25 11ú║39ú║27 _7.tif]

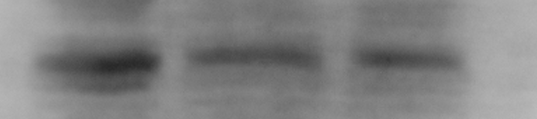

Supplement: Supplementary file 1 [file cdr-8-31-SupplementaryMaterials.zip › Western Blot/TREM1/Selected/R 2023-07-25 14ú║30ú║35 _4 .tif]

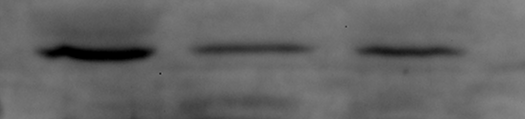

Supplement: Supplementary file 1 [file cdr-8-31-SupplementaryMaterials.zip › Western Blot/TREM1/Selected/T 2023-07-25 14ú║03ú║41 _9.tif]
