# Supplementary material for: Integrated multi-omics profiling of immune microenvironment and drug resistance signatures for precision prognosis in prostate cancer
Source: Cancer Drug Resist. 2025 Jun 25;8:31. doi: 10.20517/cdr.2025.47 (PMC12366425; doi:10.20517/cdr.2025.47)
Supplement: Supplementary file 1 [file cdr-8-31-SupplementaryMaterials.zip › cdr8047-Supplementary Figure 1.doc]

**Supplementary Figure 1.** Single-cell data quality control. (A) JackStraw results. The optimal principal components are selected based on the *P*-values on the right; (B) Elbow results. The optimal principal components generally appear at the inflection point.
